# Cross-Linked Gel Electrolytes with Self-Healing Functionalities for Smart Lithium Batteries

**DOI:** 10.1021/acsami.2c15011

**Published:** 2022-11-10

**Authors:** S. Davino, D. Callegari, D. Pasini, M. Thomas, I. Nicotera, S. Bonizzoni, P. Mustarelli, E. Quartarone

**Affiliations:** † Department of Chemistry, University of Pavia, Via Taramelli 16, Pavia27100, Italy; ‡ Department of Chemistry and Chemical Technology, University of Calabria, Via P. Bucci, Rende, Cosenza87036, Italy; § Department of Materials Science, University of Milano Bicocca, Via Cozzi 55, Milano20126, Italy; ∥ GISEL—Centro di Riferimento Nazionale per i Sistemi di Accumulo Elettrochimico di Energia, INSTM, via G. Giusti 9, Firenze50121, Italy

**Keywords:** autonomous self-healing, dynamic hydrogen bonding, smart functionalities, gel electrolyte, cross-linking, lithium batteries

## Abstract

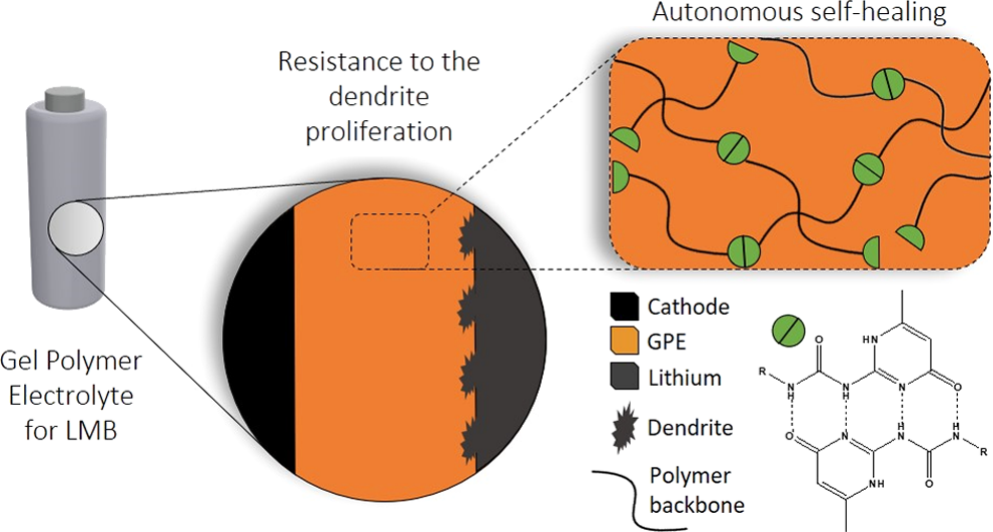

Next-generation Li-ion batteries must guarantee improved durability, quality, reliability, and safety to satisfy the stringent technical requirements of crucial sectors such as e-mobility. One breakthrough strategy to overcome the degradation phenomena affecting the battery performance is the development of advanced materials integrating smart functionalities, such as self-healing units. Herein, we propose a gel electrolyte based on a uniform and highly cross-linked network, hosting a high amount of liquid electrolyte, with multiple advantages: (i) autonomous, fast self-healing, and a promising PF_5_-scavenging role; (ii) solid-like mechanical stability despite the large fraction of entrapped liquid; and (iii) good Li^+^ transport. It is shown that such a gel electrolyte has very good conductivity (>1.0 mS cm^–1^ at 40 °C) with low activation energy (0.25 eV) for the ion transport. The transport properties are easily restored in the case of physical damages, thanks to the outstanding capability of the polymer to intrinsically repair severe cracks or fractures. The good elastic modulus of the cross-linked network, combined with the high fraction of anions immobilized within the polymer backbone, guarantees stable Li electrodeposition, disfavoring the formation of mossy dendrites with the Li metal anode. We demonstrate the electrolyte performance in a full-cell configuration with a LiNi_0.8_Mn_0.1_Co_0.1_O_2_ (NMC811) cathode, obtaining good cycling performance and stability.

## Introduction

The massive spread of Li-ion batteries in the e-mobility sector calls for a new concept of next-generation energy storage devices, which should guarantee improved lifetime, quality, reliability, and safety.^1^ All these aspects are strictly dependent on several degradation processes occurring upon continuous cycling, which involve all the cell components, such as current collector corrosion/dissolution, anode pulverization, electric contact loss, dendrite and solid electrolyte interface (SEI) growth, gas evolution, leaching of transition metals from the cathode, electrode cracks, electrode structure disordering, and electrolyte penetration.^2^

These ageing phenomena cannot be totally avoided but rather mitigated through a plethora of strategies that have been widely reviewed in the literature during the last decade.^3−7^ Very recently, breakthrough research approaches have been presented at the European level as a new roadmap, which points to developing future batteries through the integration of artificial intelligence; use of advanced tools of monitoring, evaluation, and diagnosis; and the introduction of smart functionalities such as sensing and self-healing.^8^

Self-healing is a nature-inspired concept that expresses the ability of a system to repair damage spontaneously and restore its original properties.^9^ For this reason, the development of materials for batteries capable of self-repair may be an innovative strategy to exploit higher intrinsic durability and, consequently, a prolonged cell lifetime.^10^

Self-healing functionalities have been demonstrated to pave the way for better-performing battery cells by improving the durability of materials and components. Basically, self-healing mechanisms can be divided into the following two categories: (i) intrinsic or autonomous repair of mechanical and structural cracks, chemical composition, thermal stability, and electric properties; (ii) extrinsic or nonautonomous recovery of damages triggered by external stimuli such as light, temperature, or pressure. In the specific case of Li-ion batteries, the intrinsic self-healing mechanism proved to be successful in the spontaneous repair of failed electrodes or electrolytes through reversible bond reformation in the damaged materials, reactivating their original properties.^10−13^

In the case of anodes, self-healing approaches addressed the issue of physical and mechanical damages in active materials such as silicon^14−16^ or black phosphorus^17^ induced by irreversible volume expansion and fractures upon Li (or Na) insertion/deinsertion. Recovering strategies of chemical failure were discussed for cathodes which undergo leaching of transition metals in the presence of byproducts from some Li salt degradation (e.g., LiPF_6_) or loss of small molecules such as O_2_ released from sublattice sites.^18^ Such approaches considered the use of self-healing binders based on polymers with ureidopyrimidinone (UPy) units capable of physically repairing cleavages through dynamic multiple hydrogen bonds or coating layers properly designed to trap detrimental gasses or byproducts.

Concerning the electrolyte, most of the self-healing strategies deal with solid ion conductors based on self-healing polymers capable of repairing physical damages, such as mechanical fractures or cleavages. The healing properties of solid polymer electrolytes (SPEs) with a high level of autonomous self-healing, induced by dynamic hydrogen bonding through urea groups and the disulfide metathesis reaction, which is very fast at temperatures higher than 60 °C, were reported.^19^ Highly stretchable, potentially reinforceable, and healable polymer electrolytes were also obtained by raft polymerization through a physical cross-linked network via UPy-containing brush-like poly(ethylene glycol) (PEG) chains, capable of repairing breakages at room temperature, thanks to the dynamic multiple hydrogen bonding among UPy units.^20,21^ Noncovalent π–π interactions between naphthalene di-imide and pyrene derivatives were exploited to confer tunable self-healing properties and durable ion-conductive pathways in SPEs for Li–S batteries.^22^

In this work, a novel gel polymer electrolyte (GPE) exhibiting significant self-healing capability against fracture and good transport properties is fabricated by means of *in situ* photopolymerization of poly(ethylene glycol)diacrylate (PEGDA), previously blended with ureidopyrimidinone (UPy)-telechelic, hosting a commercial liquid electrolyte. UPy-telechelic-based self-healing binders were recently proven to autonomously repair, through dynamic multiple hydrogen bonding, mechanical damages in anodes undergoing irreversible volume changes upon cycling.^17^ In contrast, gel electrolytes combining self-repairing properties with enhanced ionic transport were less explored. Few examples are obtained by loading ionic liquids within the supramolecular poly(ionic liquid) copolymer or a fully zwitterionic polymer network capable of healing cracks by exploiting hydrogen bonding.^23,24^ Similarly, gel electrolytes were produced by *in situ* polymerization of UPy-based acrylate and other acrylate monomers dissolved in proper amounts of deep eutectic solvents. In these systems, the self-healing mechanism acted on the suppression of Mn dissolution from the LiMn_2_O_4_ cathode rather than the repair of physical damage.^25^

When compared to the above-described self-healing solid polymer electrolytes, our photo-cured gel electrolyte offers additional advantages, acting as a multifunctional separator, showing (i) self-healing capability; (ii) a nanostructured network, which can potentially confine the electrodeposition of lithium to small length scales; (iii) a cross-linked backbone to increase the electrolyte moduli, enhancing the resistance to dendrite proliferation; and (iv) a chemical network contributing to immobilize the salt anion, thereby enhancing the Li^+^ transport number. Our results show that these self-healing solid-like systems are promising electrolytes for safer and more stable Li-ion and Li-metal batteries.

## Materials and Methods

### Materials

Self-healing cross-linked gel electrolytes based on PEGDA were obtained through *in situ* UV polymerization of precursor solutions. All the procedures described in the following were performed in an argon-filled glovebox (MBraun, O_2_, H_2_O < 0.5 ppm). PEGDA, cross linker, photo initiator, and the azo dye were all purchased from Sigma-Aldrich; the liquid electrolyte, LP30 (LiPF_6_ 1 M in EC/DMC 50/50 v/v), was provided by Solvionic. The UPyPEG_35000_UPy self-healing unit was synthesized as described in detail in ref (17). The UPy-acrylate monomer (UPy-MA, a comonomer shown in Figure S1) was synthesized as described in the following section.

### Synthesis of the Self-Healing Cross-Linked Gel Electrolyte PEGDA-UPy 50 and PEGDA-UPy 67

First, the precursor solution was prepared by blending a fraction of UPyPEG_35000_UPy polymer in a proper amount of liquid electrolyte LP30 (1 M LiPF_6_ in EC/DMC 50/50 v/v) to obtain two compositions with liquid concentrations of 50 wt % and 67 wt %, labeled as PEGDA-UPy 50 and PEGDA-UPy 67, respectively. This solution was stirred continuously for 20 min at room temperature and then mixed with PEGDA (*M*_n_: 700 Da), 20–22 wt % dipentaerythritol penta-/hexa-acrylate as the cross linker [containing ≤650 ppm of MEHQ (monomethyl-ether-hydroquinone) as the inhibitor], 2 wt % of Irgacure 184 (1-hydroxy-cyclohexyl-phenyl-ketone) as the photo-initiator and Sudan I dye (1-phenylazo-2-naphthol, dye content ≥95%). After vigorous stirring, the resulting homogeneous mixture was cast onto a Mylar foil with a doctor blade to obtain a wet film of 200 μm thickness and finally photo-crosslinked through two subsequent steps of UV irradiation: 5 min under a UVA lamp (365 nm) and 5 min under a UVC lamp (254 nm). The obtained GPE was then cut into round disks with 16 mm diameter and stored in a glovebox before use.

### Synthesis of the UPy-Grafted PEGDA Gel Electrolyte, UPy-g-PEGDA 67

A similar procedure was followed for the preparation of the gel copolymer electrolyte, where the UPy unit was chemically anchored to the PEGDA matrix by reaction between the acrylate groups of the two monomers, as shown in Figure S1. The UPy-acrylate (UPy-MA, comonomer *a*) was synthesized by means of the reaction between ureidopyrimidinone–iscocyanate, UPy-NCO (320 mg, 1.09 mmol), and 2-hydroxyethyl acrylate (200 mg, 1.72 mmol) in chloroform, previously dried in the presence of a catalytic amount of dibutyltin dilaurate. The reaction mixture was stirred at 60 °C under an inert atmosphere for 12 h. The mixture was cooled, filtered off to remove the excess precursor, and purified by precipitation in hexane. The precipitate was recovered by filtration, washed plentifully again with hexane, and finally dried under vacuum to obtain UPy-MA as a white powder (400 mg, yield: 90%). ^1^H NMR (400 MHz, DMSO-*d*_6_): δ 11.5, 9.64, 7.21 (br s, 3H, NH), 6.38–5.76 (m, 4H, CH_2_=CHCO acrylate and CH=CCH_3_ UPy), 4.27–4.16 (m, 4H, −OCH_2_CH_2_OCO−), 3.13–2.93 (m, 4H, CONHCH_2_−), 2.09 (s, 3H, CH_3_C=CH UPy), 1.38–1.13 (m, 8H, NHCH_2_CH_2_CH_2_CH_2_CH_2_CH_2_NH).

The UPy-MA comonomer *a* (5 equiv of the ureidopyrimidinone moiety with respect to UPyPEG_350000_UPy) was dissolved in LP30 liquid electrolyte, and the mixture was stirred for 20 min. The solution was then added to PEGDA (comonomer b), 20–22 wt % dipentaerythritol penta-/hexa-acrylate, 2% wt Irgacure 184, and Sudan I to obtain a composition with a liquid loading of 67 wt %. The precursor solution was cast and photopolymerized, as already described above.

### Cathode Preparation for Cell Assembly

The cathode slurry was prepared by using 70 wt % of active material LiNi_0.8_Mn_0.1_Co_0.1_O_2_ (NMC 811, MTI Corporation), 20 wt % conductive carbon (Timcal-Imerys, ENSACO 350P), and 10 wt % binder (polyvinylidene fluoride). The solid content of the slurry was 26 wt %. NMC811 and carbon were initially mixed in zirconia jars by a planetary ball mill at 150 rpm for 10 min, followed by a 5 min break and another 10 min of milling in the reverse direction. Subsequently, it was dispersed in a solution of poly(vinylidene difluoride) in *N*-methylpyrrolidone (NMP, Sigma-Aldrich) to obtain the slurry, which was cast on a carbon-coated aluminum foil using a doctor blade with 300 μm wet thickness. The cathode was finally dried under vacuum at 80 °C and cut into a disk of 1.6 cm diameter. The mass loading of the active material was 1.6 mg cm^–2^.

## Methods

The self-healing ability of the fractured separator was qualitatively evaluated by optical microscopy (Zeiss Olympia Axioplan). Scanning electron microscopy (SEM) analyses (both in top-view and cross-section modes) were performed using a Tescan Mira 3XMU microscope operated at 15 kV. The samples were coated with a carbon thin film using a Cressington 208 carbon coater.

Thermogravimetric analysis (TGA) of the self-healing polymers was performed by heating aliquots of about 20 mg at 5 °C min^–1^ from room temperature up to 250 °C under a N_2_ atmosphere in a Pt crucible by means of a Q5000 thermogravimetric instrument (TA Instruments, USA). Differential scanning calorimetry (DSC) analyses were performed with a Q2000 instrument (TA Instruments, USA) by heating the samples (about 20 mg) from −80 to 200 °C at 5 °C min^–1^ under a N_2_ atmosphere in Al crucibles sealed in the glovebox.

Dynamic mechanical analysis (DMA) measurements were performed on rectangle-shaped samples (35 mm × 10 mm) by a Metravib DMA/25 equipped with a shear jaw for films. The frequency sweep experiments were carried out in the frequency range between 0.2 and 20 Hz at a constant strain of 1 × 10^–3^ %, from 20 to 80 °C every 10 °C. Temperature sweep tests were performed at a heating rate of 3 °C, over a range between 20 °C and 120 °C, at a dynamic stress amplitude of 1 × 10^–3^, and a frequency of 1 Hz.

^1^H NMR high-resolution spectra were recorded on a Bruker 400 MHz instrument. Solid-state NMR data were collected for pristine and cycled separators on an AVANCE III Bruker 400 MHz spectrometer (9.4 T magnet) using a 4 mm MAS probe. ^13^C spectra were acquired with the ^13^C–^1^H CP-MAS sequence under the same MAS conditions. The ^1^H π/2 pulse was 2.5 ms, the delay time was 5 s, the contact time was 2.5 ms, and the signals were averaged over 8k acquisitions. Quantitative ^13^C spectra were acquired under ^1^H high-power decoupling conditions with a π/2 pulse of 4.7 μs, a recycle delay of 40 s, and a SPINAL-64 heteronuclear decoupling scheme. ^13^C chemical shifts were referred to adamantane as a secondary standard with respect to tetramethylsilane (TMS, 0 ppm). ^31^P spectra were collected at room temperature and 75 °C with one-pulse sequence under MAS conditions at 10 kHz, with a delay time of 30 s, π/2 pulse of 4 ms, and averaging over 32 scans. The ^31^P chemical shifts were referenced to aqueous 85% H_3_PO_4_ solution (0 ppm). ^7^Li spectra were obtained both under static and MAS conditions at 10 kHz in the temperature range of 25 to 75 °C. Static spectra were acquired with one-pulse sequence using a 5 s time delay, π/2 pulse of 3.5 ms, and averaging over 64 scans. MAS spectra were acquired with a 5 s time delay, π/2 pulse of 3.5 ms, and averaging over 128 scans. ^7^Li spin-lattice relaxation times (*T*_1_) were measured with the use of a standard inversion recovery pulse sequence, under static conditions, in the same temperature range. The rotors were filled in an Ar-filled glovebox (H_2_O < 0.1 ppm and O_2_ < 0.1 ppm) to prevent degradation of the samples. The spectra were acquired, processed, and analyzed with the software package Topspin 3.1 (Bruker).

The ionic conductivity was measured between −10 and 70 °C by means of electrochemical impedance spectroscopy (EIS), using a frequency response analyzer (Solartron 1255) connected to an electrochemical interface (Solartron 1287), by applying an AC voltage of 50 mV in the frequency range between 1 and 10^5^ Hz.

The Li transference number, *t*_Li^+^_, was calculated by coupling EIS and chronoamperometry experiments on a Li|electrolyte|Li symmetrical cell, as defined by the Bruce–Evans equation^26^
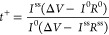
where Δ*V* is the applied voltage (15 mV), *I*^0^ and *I*^ss^ are the current densities at the beginning of the polarization and at the steady state, respectively, and *R*^0^ and *R*^ss^ are the interfacial resistances before and after polarization, respectively.

Linear voltammetry was carried out to determine the electrochemical stability window (ESW) of the electrolyte by means of an electrochemical interface (Solartron 1287) in the voltage range of −1 to 6 V, with a scan rate of 0.25 mV s^–1^. A two-electrode cell was used with lithium metal as both the counter and reference electrodes, and carbon-coated aluminum as the working electrode. The Li electrodeposition was studied by means of galvanostatic stripping/plating experiments performed at room temperature on Li|electrolyte|Li symmetric 2032-type coin cells by a battery tester (Arbin, model BT-2000). The cell was periodically cycled (1 h per cycle) at fixed current densities ranging between 0.01 and 0.05 mA cm^–2^. The NMC/Li full cells were cycled on a Biologic BCS-810 battery tester from 3 to 4.3 V, with the C rate ranging between C/10 and C/2 (C/2 corresponding to a current density value of 0.154 mA g^–1^). All the potentials reported refer to the Li^+^/Li couple. The impedance on the cells was measured by means of EIS at room temperature by applying an AC voltage of 50 mV in the frequency range of 0.1 Hz to 1 MHz. For the sake of comparison, similar electrochemical experiments were also carried out on control cells, including the LP30 solution as the liquid electrolyte supported by a Whatman glassy fiber separator (GF/C).

## Results and discussion

### Structure of the Cross-Linked Network of Gel Electrolytes

**Figure 1:**
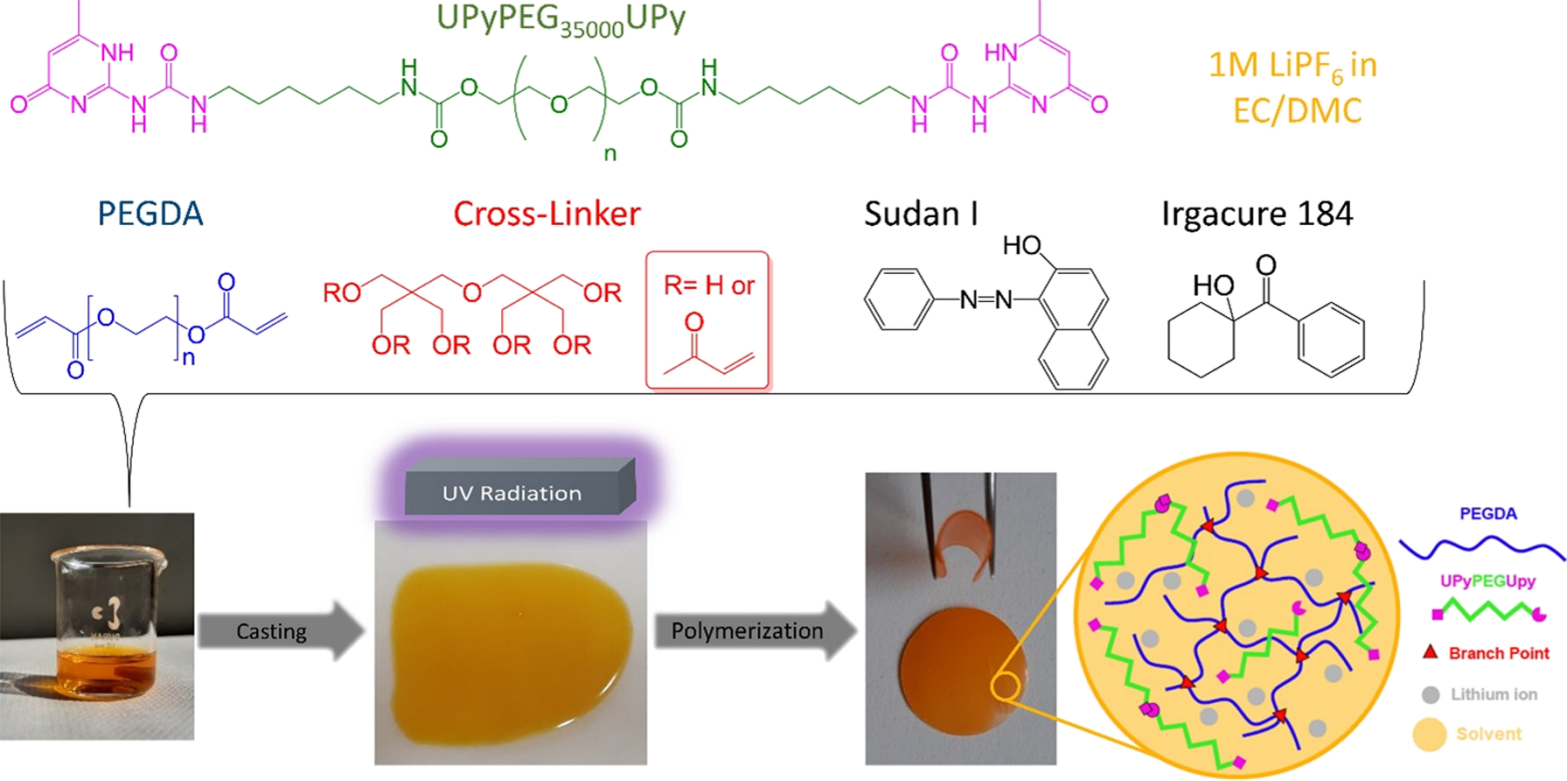
Synthesis of cross-linked gels PEGDA-UPy *X* (*X* = 50 and 67 wt %).

Two self-healing cross-linked gels, with different hosted liquid electrolytes, namely PEGDA-UPy 50 (LP30 = 50 wt %) and PEGDA-UPy 67 (LP30 = 67 wt %) respectively, were fabricated by *in situ* photoinitiated free-radical polymerization via a fast one-step process, as depicted in Figure 1.

No chemicals other than the network precursors were used to avoid undesired traces of impurities, such as solvents. To this aim, PEGDA was blended with the self-healing unit (UPy-PEG telechelic), mixed with the cross linker (dipentaerythritol penta-/hexa-acrylate), initiator (Irgacure), absorber (Sudan) and liquid electrolyte (LP30). The resulting homogeneous mixture was finally filmed under UV curing after being cast with a spacer at a defined thickness. After a concentration survey of the cross linker between 5 and 25 wt %, the optimal value of 20 wt % was chosen for both the gels, which allowed us to achieve an efficient and uniform network, while not making it too brittle, and capable of retaining the entrapped liquid electrolyte.

The cured gels were first characterized by FTIR spectroscopy to confirm the disappearance or, at least, the drastic reduction of the acrylate and vinyl double bonds at about 1620 and 1640 cm^–1^, respectively, typical of PEGDA (Figure S2).^27^ The analysis of such bands in terms of intensity and peak area allowed us to roughly estimate a conversion degree of about 70%.

**Figure 2:**
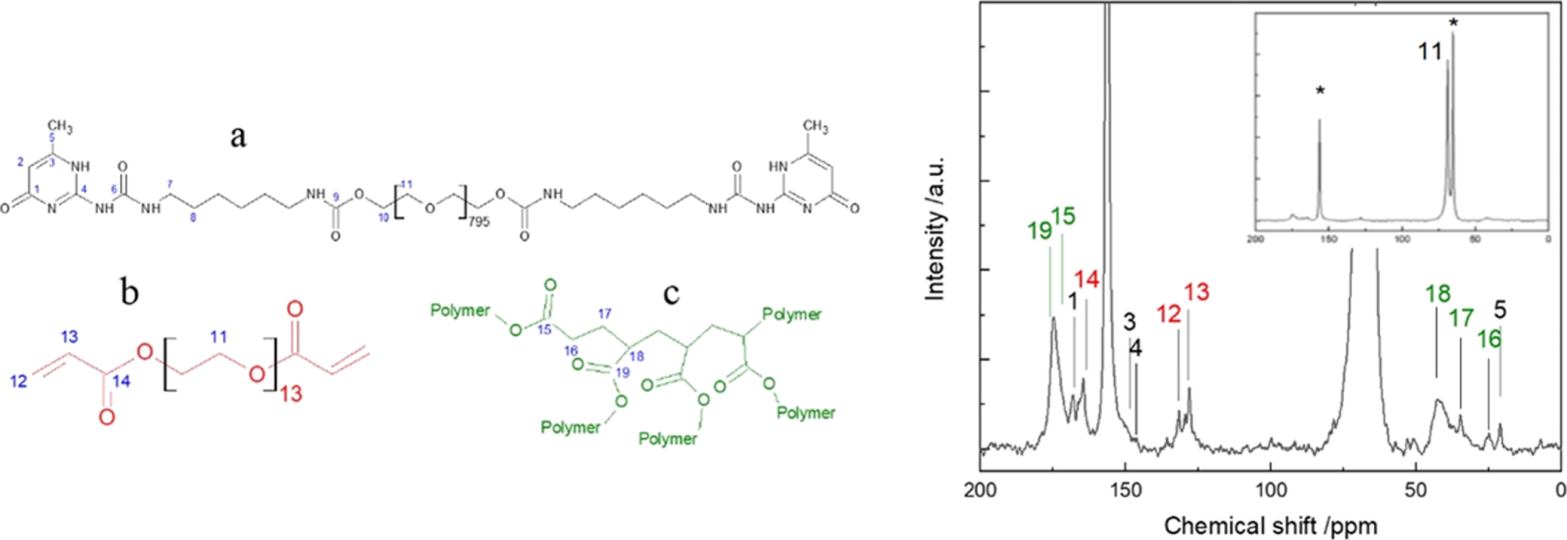
(Right) ^13^C MAS-NMR spectrum of the pristine sample and peak assignment. The peaks marked with stars at ∼70 and ∼160 ppm refer to the −CH_2_ and −C=O groups of LP30 liquid electrolyte, respectively; (left) peak assignment of UPyPEG_35000_UPy (a), PEGDA (b), and the “ziplike” network originating from *in situ* polymerization (c).

Solid-state NMR was used to check the conversion degree of the PEGDA vinyl and acrylate groups and to provide an estimation of the network cross-linking density. Figure 2 shows the ^13^C MAS-NMR spectrum of the pristine sample obtained under high-power ^1^H decoupling conditions to obtain quantitative information. The spectrum is dominated by the signals of the electrolyte solvents (EC and DMC, see figure caption) and of the ether carbon groups of the polymer chains. However, the minor resonances can also be clearly observed and assigned.^28,29^ The degree of cross-linking due to PEDGA photopolymerization and the average number of brushes, or nodes, can be determined by considering the relative amounts of unreacted vinyl groups (assignments 12 and 13 in the PEGDA scheme (b)) and of carbonyl species in the reacted “ziplike” network [15 and 19 in scheme (c)]. The analysis reveals a branching ratio of about 3 and a degree of polymerization of 70%, in excellent agreement with the FTIR data. The computation details are reported in the Supporting Information (Figure S3).

**Figure 3:**
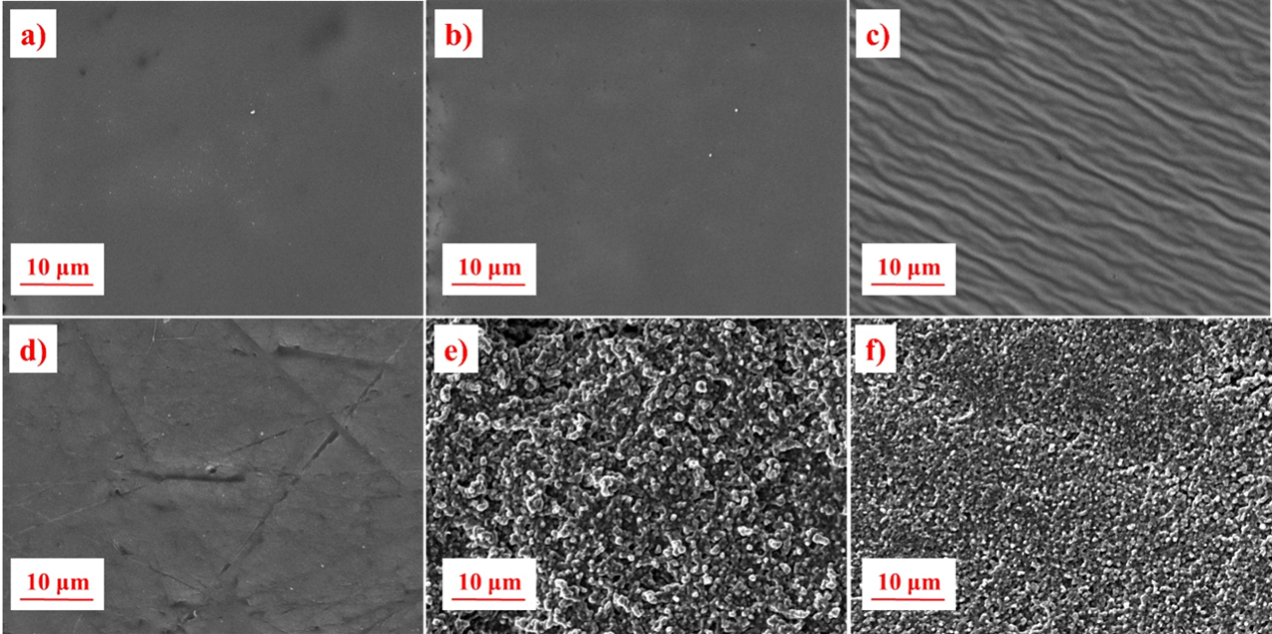
SEM images in top-view (upper) and cross-section (down) modes of PEGDA (a and d), PEGDA-UPy 50 (b and e), and PEGDA-UPy 67 (c and f).

Figure 3 reports the SEM images both in top view (a–c) and in cross-section (d–f). The image of the cross-linked polymer without any hosted liquid electrolyte is also reported for the sake of comparison. The gel films show a solid-like morphology, made of spherical-shaped polymeric globules, tightly packed, larger in the case of PEGDA-UPy 50. No cavities eventually filled by the free liquid component are detectable, and this evidence would indicate that the electrolyte solution swells the polymer chains during the polymerization, rearranging them in capsule-like domains. The top-view images (Figure 3b,c) provide evidence that the PEGDA-UPy 50 displays a much flatter morphology with respect to PEGDA-UPy 67, whose higher roughness is related to the higher concentration of liquid hosted by the network.

**Figure 4:**
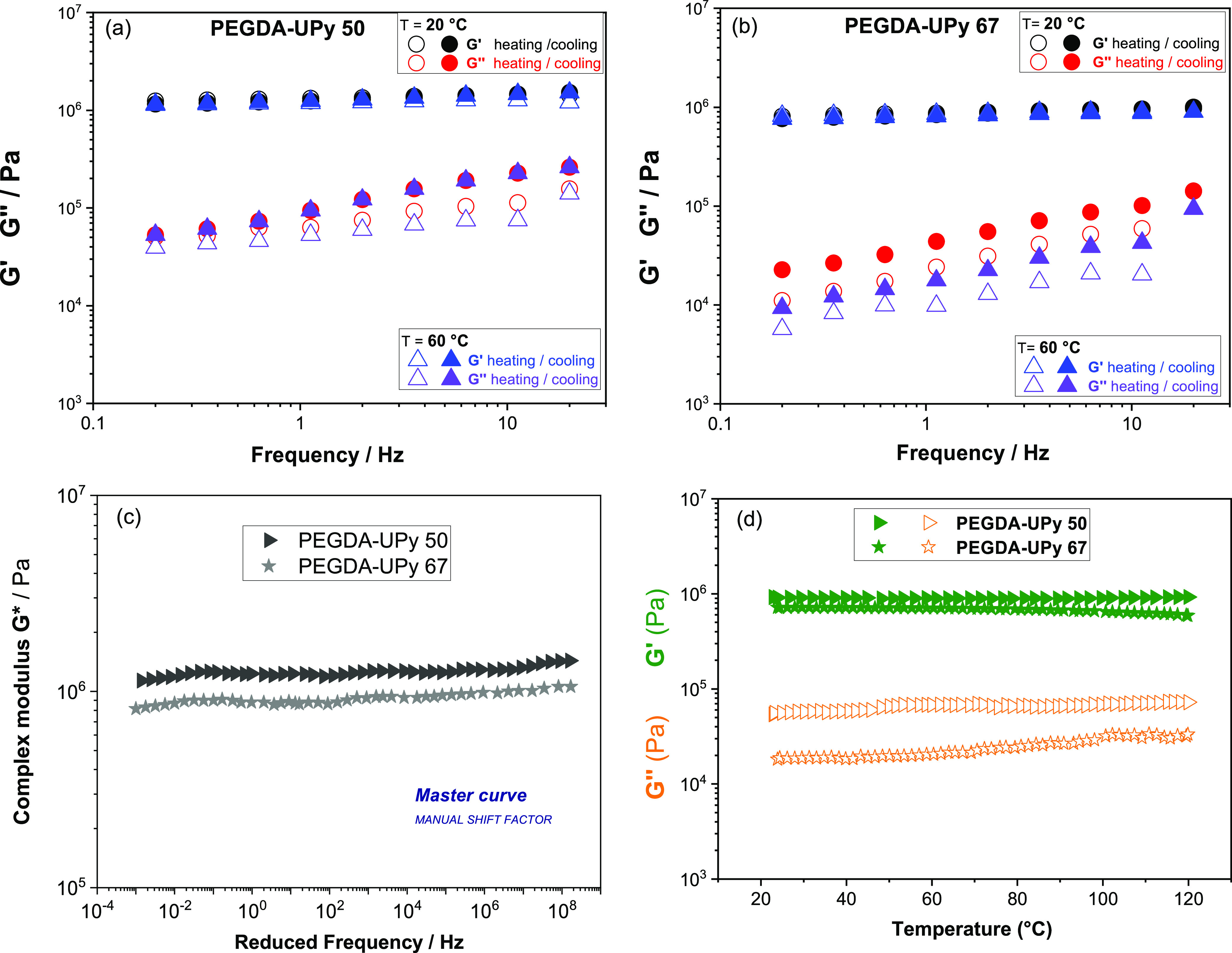
Frequency sweep test (a and b), master curve (c), and temperature sweep test (d) of the two samples PEGDA-UPy 50 and PEGDA-UPy 67.

DMA measurements were performed in a temperature range between 20 and 70 °C to measure the elastic storage, *G*′, and the viscous moduli, *G*″, of the two cross-linked gels, as a function of temperature and oscillation frequency. Figure 4 shows the moduli behavior in the frequency sweep tests at two representative temperature values, 20 and 60 °C, for PEGDA-UPy 50 (a) and PEGDA-UPy 67 (b), respectively. In both cases, *G*′ is independent of the frequency and temperature and is about 100 times higher than *G*″, achieving values of 1.5 and 0.8 MPa for PEGDA-UPy 50 and 67, respectively. The difference between *G*′ and *G*″ is a good index of the elastic properties of the gel electrolytes, which is an important feature required in the design of autonomous self-healing system. Such values are in very nice agreement with those reported for other PEGDA-based cross-linked gels, photocured by similar strategies, and even for cross-linked solid polymer electrolytes (namely without any hosted liquid within the network).^30^ This evidence could be an index of high uniformity of the cross-linking (high conversion degree and few free/dangling chains), which is not altered by the presence of a liquid electrolyte inside the membrane. In addition, tests performed in a heating/cooling scan show that these gels do not lose elasticity (*G*′ values are overlapped) over thermal cycles.

The extension of the frequency range of investigation of the modules can be realized by means of the so-called time–temperature superposition principle (tTS), which allows us to overcome the instrumental limits concerning the explorable frequency range (i.e., from 0.2 to 20 Hz). The tTS procedure is based on the principle of time–temperature equivalence, that is, the general behavior of a material sheared over enough time to flow or heated to soften. It was applied to frequency sweep tests performed at various temperatures in order to build the master curves of the complex module G* (G* = *G*′/*G*″), choosing the reference temperature as 40 °C and extending the frequency window from millihertz to hundreds of megahertz (Figure 4c). The mechanical spectra we observe further emphasize the typical solid-elastic behavior of these polymer films.

Finally, the temperature sweep tests (Figure 4d) performed on both gels from 20 to 120 °C, confirm the invariability of the elastic modulus *G*′ over a wide temperature range. As also observed in the frequency tests, PEGDA-UPy 50 has not only a higher elastic modulus than PEGDA-UPy 67 but also a higher viscous modulus *G*″. This supports the fact that the addition of a larger amount of electrolyte solution does not weaken the mechanical properties of the final film because a good and uniformly cross-linked solid electrolyte was created.

By assuming such network uniformity, the modulus values obtained by the rheological measurements may be used to determine some parameters describing the cross-linked polymer network, such as (i) the molar mass, *M*, of the network chains between the chemical crosslinking and chain entanglements and (ii) the mesh size or correlation length, *d*, which may be considered as the distance between the cross linkers or branch points in the gels. These parameters were determined by the following equations^30−32^
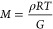

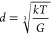
where ρ is the PEGDA density (1.12 g cm^–3^), *R* and *k* are the gas and Boltzmann constants, respectively, *T* is the temperature, and *G* is the storage modulus at high frequencies. The *M* value of 1820 g mol^–1^ was calculated for PEGDA-UPy 67 and *M* = 3636 g mol^–1^ for PEGDA-UPy 50. Despite of the different molecular weight of the chains among the branching points, the estimated mesh sizes of the gels are similar, namely 1.4 and 1.7 nm for PEGDA-UPy67 and PEGDA-UPy 50, respectively. This evidence is in good agreement with the literature, which states that *d* is independent of *M* but strictly related to the cross-linking density of the polymer.^31^ In such specific systems, the determined average mesh sizes indicate that both the networks are tightly cross-linked, as expected from the conversion degree estimated from NMR and IR.

The gel’s thermal properties were investigated by TGA and DSC, whose traces are reported in Figures S4 and 5a, respectively. The electrolytes are thermally stable, also in terms of electrolyte evaporation, at least up to 100 °C, contrary to the pristine LP30 solution, for which a weight loss >15 wt % is noticed as early as at 50 °C. DSC plots provide evidence of the effect of the cross-linking process on the thermal properties of the whole gel. As noticeable by a comparison between liquid and gels, the curing enhances the melting and crystallization temperatures of the liquid electrolyte in the polymer, likely due to its tight confinement within the polymer network. This phenomenon is especially evident in the case of PEGDA-UPy 67, which hosts a higher LP30 concentration. On the other hand, similar glass-transition temperatures, *T*_g_, are observed at about −42 °C (Figure S5), indicating good chain mobility around room temperature, which is fundamental to boosting the self-healing functionalities via multiple dynamic bonding.

**Figure 5:**
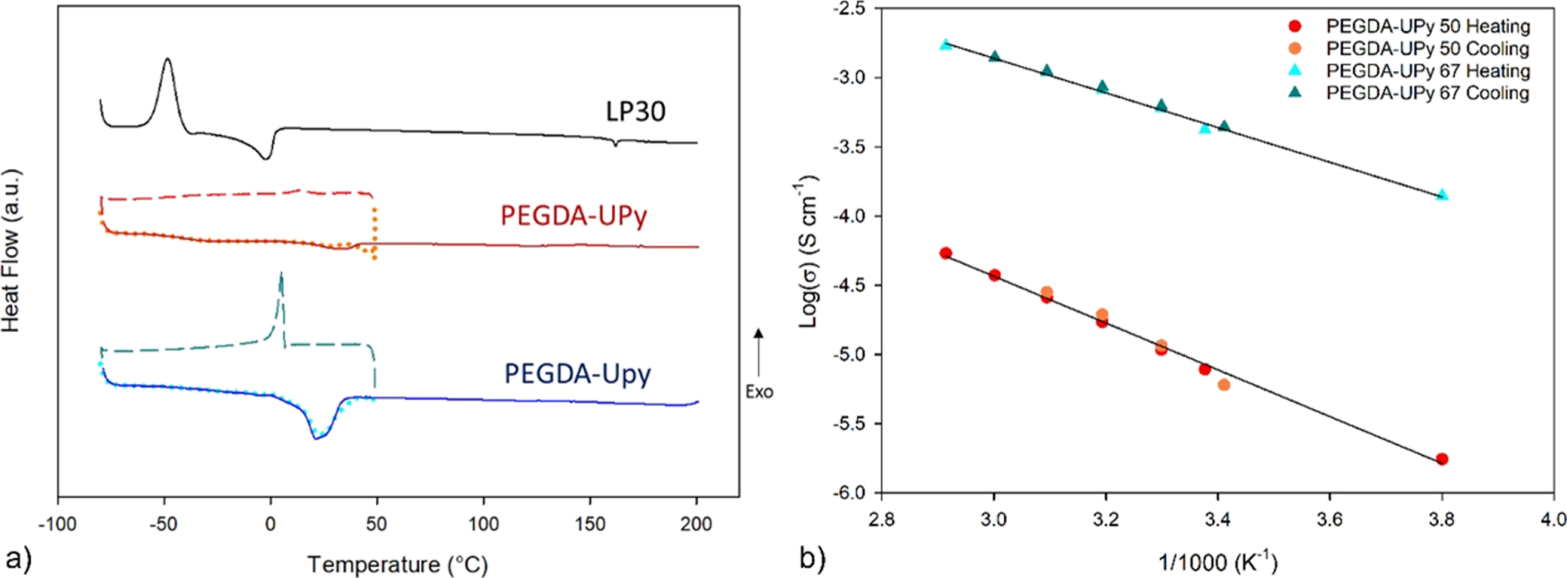
(a) DSC analysis of liquid electrolytes LP30 (black line), PEGDA-UPy 50 electrolyte (red line), and PEGDA-UPy 67 (blue line). Scan from −80 to 50 °C (dotted line), scan from 50 to −80 °C (dashed line), and scan from −80 to 200 °C (solid line). (b) Conductivity *vs* temperature, *T*, for PEGDA-UPy 50 and PEGDA-UPy 67 in the range of −10 to 70 °C.

### Li^+^ Transport Properties

Temperature-dependent ionic conductivity was investigated by means of EIS within the range of −10°–70 °C on both the PEGDA-UPy 50 and PEGDA-UPy 67 gels. As shown in Figure 5b, the data were found to obey the simple Arrhenius model, with very good reproducibility in two heating/cycling cycles, owing to the absence of thermal phenomena, as confirmed by DSC. The calculated activation energies for the ion hopping were 0.33 and 0.25 eV, respectively, in good agreement with what were observed for other cross-linked solvate gels.^24^ PEGDA-UPy 67 showed good ionic conductivity, exceeding 1.0 mS cm^–1^ at 40 °C, which is about 2 orders of magnitude higher than that measured for PEGDA-UPy 50. The differences in the conductivity behavior imply that the amount of polymer in PEGDA-UPy 67 were such that they allowed ion transport predominantly occurring through the liquid phase, contrary to PEGDA-UPy 50, where the amount of liquid electrolyte was lower, and the network became more effective in constraining local motions, eventually assisted by ether-based chains.^33^ Based on such results, PEGDA-UPy 67 was considered a better electrolyte, combining liquid-like transport with solid-like mechanical properties. This composition was selected for further electrochemical characterization and assembly of full cells.

Li-ion transport number (*t*_Li^+^_) was measured at room temperature by coupled chronoamperometry/impedance techniques (Figure S6).^26^ A *t*^*+*^ number of 0.6 was determined for the gel, which is higher by about a factor of 2 than that of pristine liquid electrolyte. The enhancement in the cation fraction contributing to the ionic transport has been often observed in literature in the case of active hosting scaffolds, both polymeric and inorganic.^24,34^

**Figure 6:**
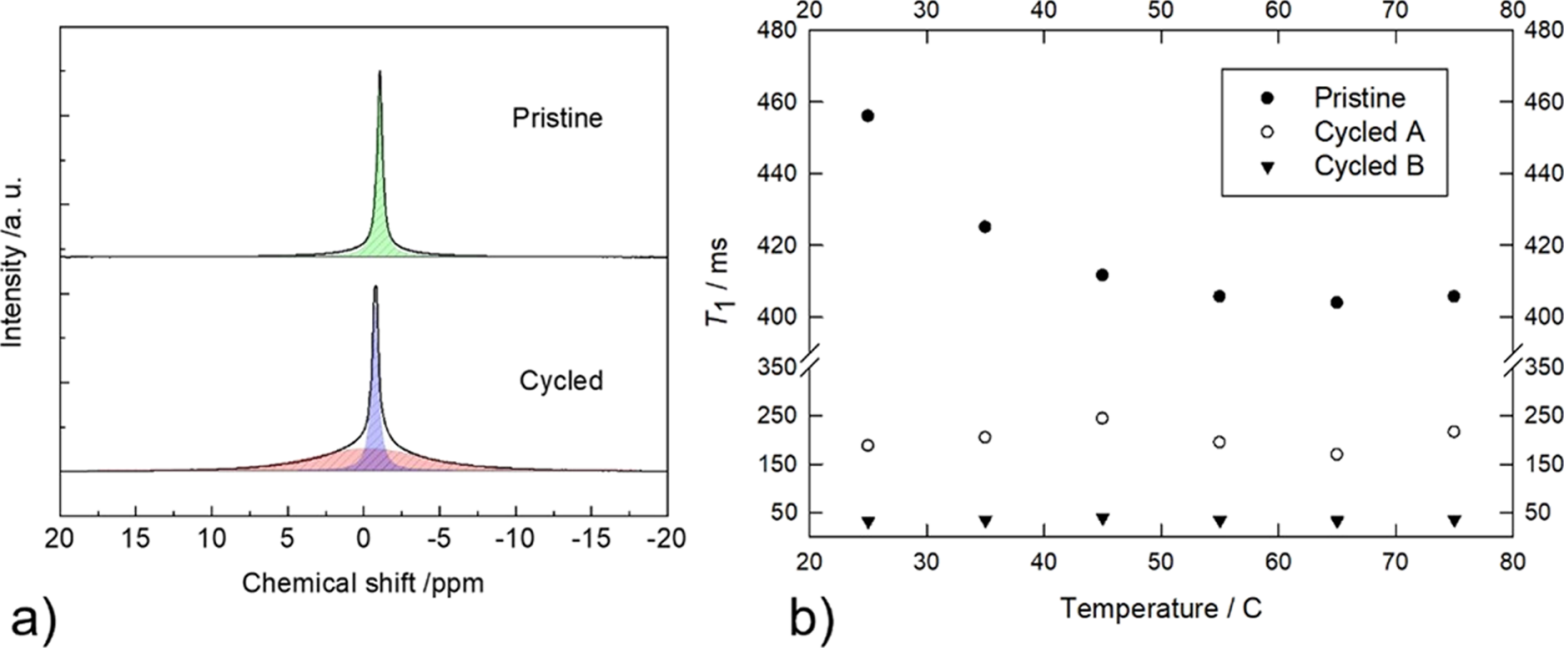
(a) ^7^Li MAS-NMR spectra of the pristine (top) and cycled (bottom) samples and (b) *T*_1_ relaxation times *vs* temperature. In the cycled sample, populations A and B are highlighted in blue and red, respectively. The cycled sample refers to the gel recovered after galvanostatic cycling of the Li|gel|NMC 811 full cell (see the pertinent section).

Figure 6a compares the ^7^Li MAS-NMR spectra of the pristine gel and of the same sample (named “cycled”) after galvanostatic cycling was carried out on the Li|gel|NMC full cell (see below). The pristine sample shows a single line centered at −1.1 ppm, which means that all the lithium ions are experiencing the same chemical environment, in agreement with previous literature.^28,34^ The best-fit NMR parameters of the peak are reported in Table 1. In particular, the peak has a full Lorentzian shape, as expected for a highly mobile species. In fact, the behavior *versus* temperature of the spin-lattice relaxation time, *T*_1_, reported in Figure 6b shows that the spin population is near to the motional narrowing regime, which is reached at about 60 °C at the minimum of the *T*_1_ curve.^35^ The motional narrowing regime can be defined as the region where the relationship
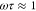


**Table 1:** Best-Fit Results of the ^7^Li MAS-NMR Spectra of Figure *n* – 1^a^

	FWHH (Hz)	*xG*/(1 – *x*)*L*	chemical shift (ppm)	abundance (%)
pristine	78	0.00	–1.1	100
cycled A	82	0.00	–0.8	37
cycled B	1386	0.65	–0.3	63

a FWHH = full width at half height; *xG*/(1 – *x*)*L* = Gaussian/Lorentzian ratio.

holds, where ω is the Larmor frequency of the nuclei, and τ is correlation time for spin motion.^36^ At every temperature, the ^7^Li nuclear relaxation can be fitted with a single exponential (see Supporting Information, Figure S7), as expected for a population of equivalent spins.

In contrast, the ^7^Li spectrum of the cycled sample shows the presence of two populations, called A and B (see best fits of Figure 6a), whose NMR parameters are reported in Table 1. Population A is again characterized by a full Lorentzian peak shape with nearly the same line width as that of the pristine sample. In contrast, population B shows a much greater line width of prevalent Gaussian nature, which calls for reduced mobility and/or higher static disorder (see Table 1). The spin-lattice relaxation curves of the cycled sample cannot be fitted with a single exponential but require two exponentials for a correct fitting (see Figure S8). The two populations have different mobility and/or interaction strengths with the lattice, as the *T*_1_ values scale between them roughly by a factor of five (see Figure 6b). In addition, the *T*_1_ values of population A of the cycled sample are shorter than those of the pristine one, which points toward a more efficient relaxation process. This could be due to the presence of paramagnetic impurities due to battery cycling (e.g., graphite, carbon black, transition metals). Interestingly, both populations show nearly constant *T*_1_ values *versus* the temperature, which indicates they are both in the motional narrowing regime. These overall results can be explained by assuming that the cycled sample is characterized by a greater structural disorder that accounts for the greater isotropic distribution of chemical shifts and by additional terms in the density of states of the spin-lattice relaxation mechanisms.

### Self-Healing Ability

**Figure 7:**
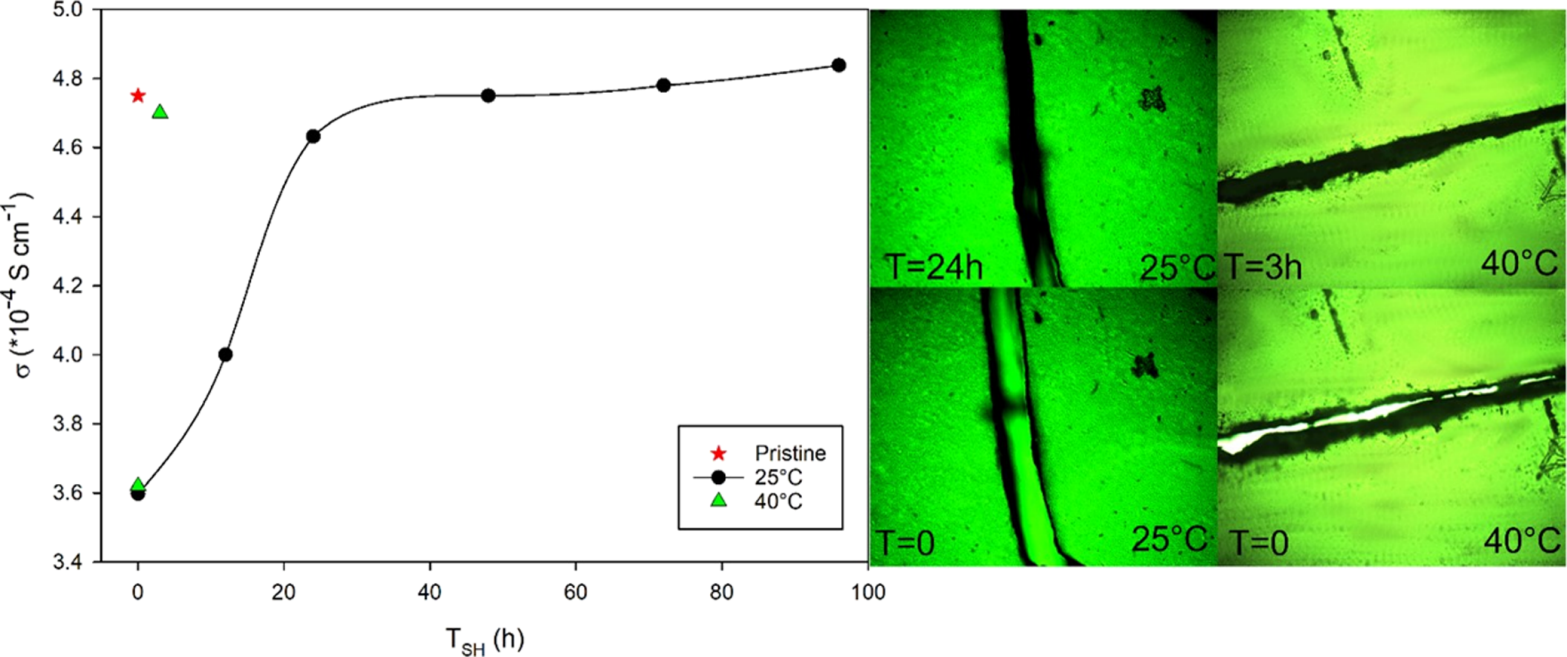
Ionic conductivity and optical microscopy images of PEGDA-UPy 67 measured at 25 and 40 °C before and after applying a deep fracture with a blade (100% of the whole film thickness).

The self-healing capability of the cross-linked gels was conferred by introducing a UPy-based telechelic (UPy-PEG-UPy) within the network (see Figure 1), which was demonstrated to have outstanding repairing properties against mechanical damages by means of dynamic multiple hydrogen bonding among the ureidopyrimidinone moieties.^17^ To evaluate the effects of the mechanical fracturing and the subsequent spontaneous healing on the ionic conductivity of the gel electrolyte, the PEGDA-UPy 67 film was deeply cut over the whole thickness. Figure 7 shows that the ionic conductivity is reduced after applying a deep crack; however, more than 95% of the pristine value was recovered after 20 h at 25 °C. The self-healing kinetics is significantly improved with the temperature. Indeed, the time required to repair the fracture is significantly reduced when the gel is damaged, resulting in full crack healing after only 3 h at 40 °C.

To investigate in more detail the self-healing mechanism of the UPy-based unit, a different synthetic approach was followed based on the direct reaction between PEGDA and UPy-including monomer (UPy-MA comonomer *b* in Figure S1). This reaction leads to a gel (PEGDA-g-UPy 67, with the same concentration as the hosted liquid, 67 wt %), where the self-healing moiety is directly grafted onto the polymer backbone instead of being homogeneously blended as in PEGDA-UPy 67. Figure S9 reports the behavior of room-temperature conductivity with time after the deep cracking. It is noteworthy that there was no healing of the membrane fracture even after 40 h of storage, but the electrolyte underwent full breaking, leading to cell short circuiting. The self-healing inability of such a system is likely due to strong grafting, leading to a rigid network, where the inter-/intrachain motions necessary to boost the dynamic hydrogen bonding required for self-healing were strongly hindered.

### Electrochemical Performances

An electrochemical stability window higher than 5.5 V was evaluated for the PEGDA-UPy 67 gel by means of linear voltammetry, which is about 1.5 V wider than that reported for the pristine liquid electrolyte.^34^Figure S10 shows the cathodic and anodic branches of the potentiodynamic test: no significant oxidizing decomposition phenomena are detected up to 5.5 V *versus* Li/Li^+^; similarly, only minimal breakdown is evident around the onset of the Li plating. Even though the terminal H-bonds and urethane units are present in the backbone, the polymer matrix plays a positive role in the enlargement of the GPE electrochemical stability window. Indeed, the UPy telechelics based on quadruple hydrogen bond arrays are very stable from a chemical and thermodynamic point of view, thanks to the very strong and directional interactions and the self-complementary nature of the ureidopyrimidinone functional group (UPy).^37^ For this reason, the high electrochemical stability is comparable to what is usually obtained in the case of solid-state electrolytes, despite the large presence of liquid (>60%) in the GPE.

**Figure 8:**
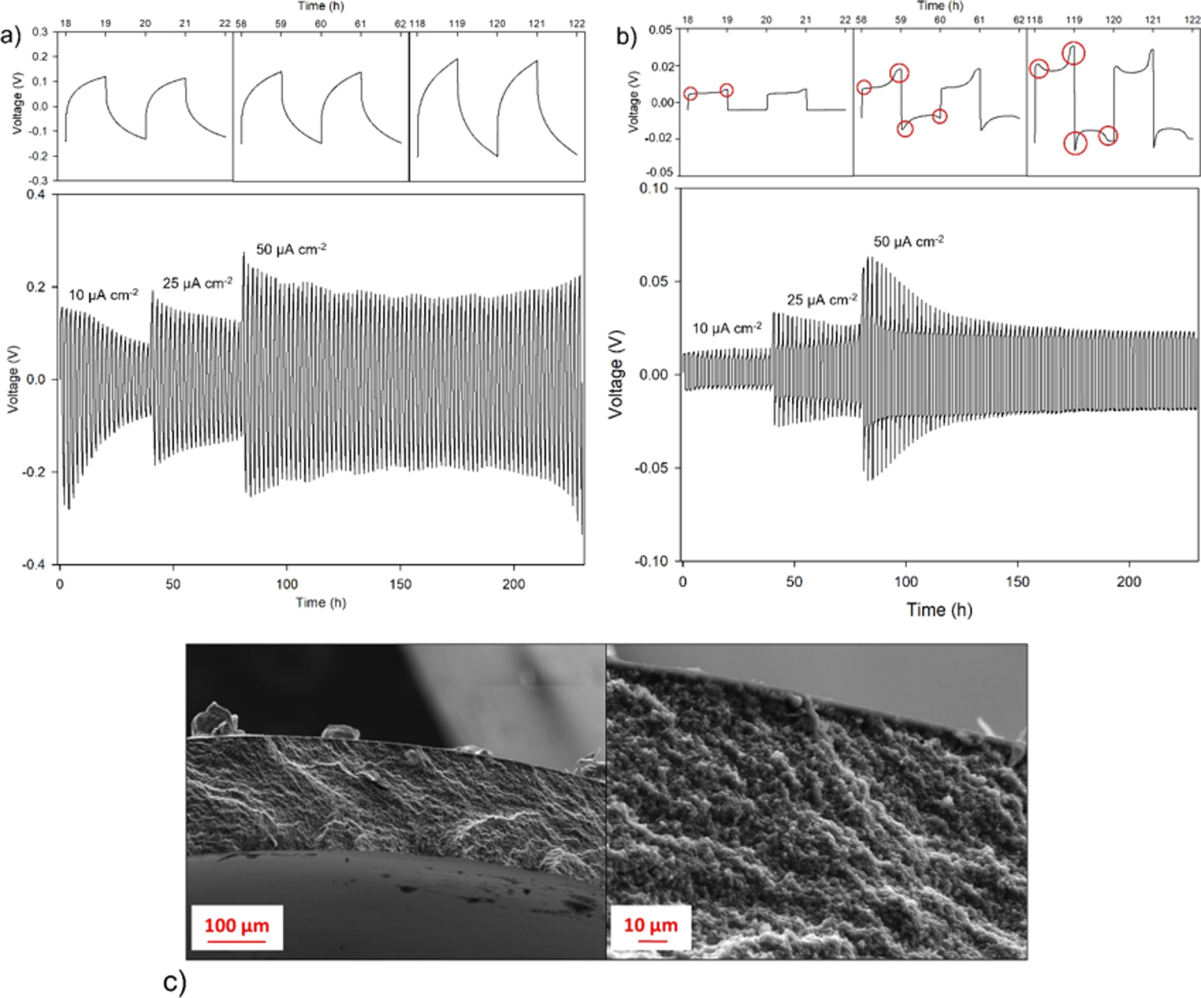
Galvanostatic stripping and plating experiments on symmetric Li|PEGDA-UPy 67|Li (a) and Li|LP30|Li (b) cells; (c) SEM images showing the cross-sectional view of the gel after the galvanostatic electrodeposition experiments.

Preliminary galvanostatic stripping and plating experiments, reported in Figure 8a, were carried out at room temperature on a Li/Li symmetric cell with PEGDA-UPy 67 as the electrolyte at different current densities, for example, 10, 25, and 50 μA cm^–2^. The cycling process was compared to that obtained for the liquid electrolyte supported by a glass fiber separator (Whatman, GF/C), whose plot is shown in Figure 8b.

As expected, the cross-linked gel exhibits higher overpotential than the liquid electrolyte due to a difference in Ohmic resistance. The polarization potential could be in principle decreased by reducing the electrolyte thickness or further improving the Li-ion interfacial transport and local interface environment.^38^ However, the liquid system shows significant potential drift at higher current density and exhibits the typical unstable profiles associated with pitting (red cycles in Figure 8b) and stripping of lithium from electrochemically disconnected mossy deposits. The pitting phenomena are absent in the case of the gel electrolyte, whose voltage profile rather resembles an arc-like shape, usually due to a more tortuous pathway for Li-ion transport typical of the solid and solid-like systems. Such results would suggest that highly cross-linked gels may effectively prevent mossy lithium deposits, which are responsible for the dendrite propagation and unstable Li electrodeposition.

The mitigation role of the gel against dendrite proliferation may be explained by considering the structure of the cross-linked network used as the electrolyte, consisting of a backbone with high cross-linking density (related to the gel’s nanometric mesh size) and solid-like storage moduli, which are able to partially immobilize the anion of the Li salt. These properties are crucial factors for stabilizing the electrodeposition of highly reactive metals. Archer’s group described how the morphological instabilities during the Li stripping and plating can be reduced by replacing the liquid electrolyte with polymer-based separators, where a good interplay is possible between mechanical properties and ion transport.^39^ Whereas, it is believed that dendrite suppression occurs when the electrolyte/separator has an elastic modulus similar to that of the metal (i.e., on the order of a few gigapascals for Li),^40^ significant improvements in lithium electrodeposition may be obtained even at much lower storage moduli (few megapascals), if the polymer electrolyte is capable of immobilizing at least a small fraction of anions to avoid the formation of large electric fields near the lithium electrode, which are responsible for the undesired preferential deposition as dendritic tips. Even if the storage modulus is about 1 MPa (far from what was predicted by Newman and Monroe for an ideal resistance to dendrite proliferation^40^), the uniform and highly cross-linked PEGDA-UPy 67 with nanometric mesh size has a high *t*_Li^+^_ (0.6); these interplaying properties may justify why the perturbation growth is slowed and no pitches are observed, at least in the used electrochemical conditions. This is in nice agreement with the literature and especially with the model predicted by Archer and coworkers in the case of separators with moduli on the order of 10^6^ Pa and a fraction of immobilized anions of about 0.5.^39,41,42^ This result is confirmed by the SEM images of the gel (Figure 8c) and of the Li electrode surface (Figure S12), which were collected after the galvanostatic stripping/plating experiments. It is evident that the surface of both the gel electrolyte and Li electrode does not show metallic deposits due to mossy lithium.

The impedance evolution during the Li stripping and plating experiments carried out on the symmetric Li|GPE|Li was also used to evaluate the interfacial stability of the electrolyte against the Li metal anode. Figure S11 shows the Nyquist plots collected on such a cell at *t* = 0 before the electrodeposition experiment and after 230 h of cycling at room temperature. Small increase in the interfacial resistances (determined by the semicircle diameter) demonstrates that the PEGDA-UPy 67 gel has an overall good compatibility with Li electrodes.

**Figure 9:**
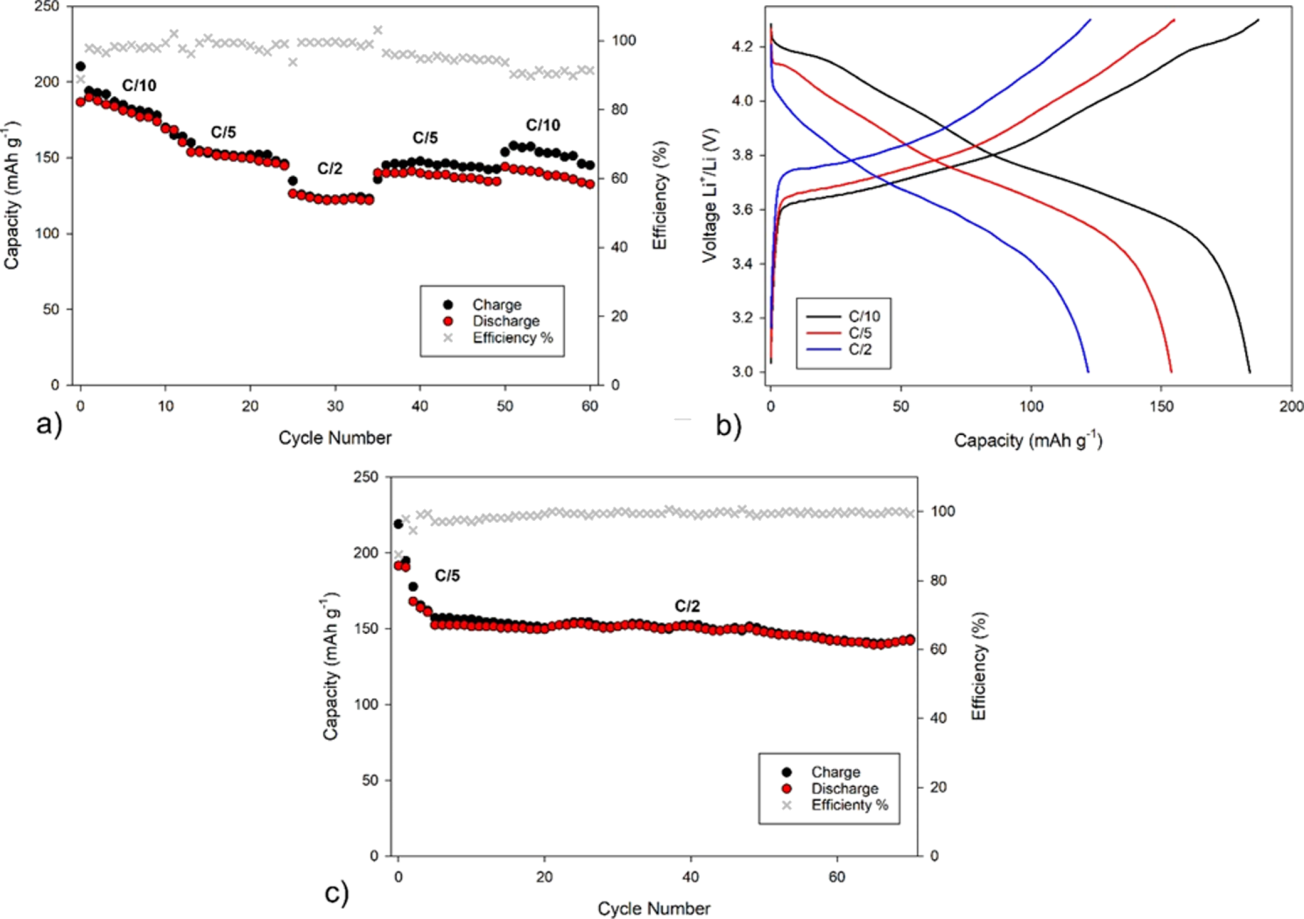
Cycling performance of the Li|PEGDA-UPy 67|NMC811 (black and red circles) full cell at room temperature between 3.0 and 4.3 V. (a) Rate performance at different C rates; (b) voltage profiles at C/10, C/5, and C/2 for the GPE-based cell; (c) galvanostatic cycling at C/2 after two equilibration cycles at C/5.

PEGDA-UPy 67 was finally tested in a cell Li|GPE|NMC811. Figure 9a–c reports the preliminary cycling behavior of the self-healing cell, namely the voltage profiles at C/10, C/5, and C/2 (Figure 9a), the rate performance (Figure 9b), and the cell stability at C/2 (Figure 9c).

The cross-linked gel shows very good functional performance, offering significant advantages in terms of interface stability, electrochemical stability window, and self-healing capabilities. Some overcharge phenomena are evident in the case of cycling back @0.1 C at the end of the rate performance test. They may be observed in the case of half cells when the amount of cathode materials is very low with respect to the anode and are likely due to partial degradation of SEI or/and cathode active material (NMC) upon cycling.

Good active material utilization and capacity retention are also observed in the explored cycling range, at a moderate C rate (C/2) over more than 70 cycles with a capacity retention >95%, a Coulombic efficiency of 99%, and a delivered specific capacity equilibrated at about 150 mA h g^–1^. The capacity fluctuation during cell cycling has already been observed in the literature for similar systems, and interpreted in terms of gradual equilibration of the ionic transport at the interface level.^30,33,43^ This is confirmed by the decrease in the interface resistance observed by means of EIS on the full cell after galvanostatic cycling, as shown in the Nyquist plots reported in Figure S13. Our future investigations to improve the performance and capacity will include optimization of the cathode composition and preparation, gel polymerization directly onto the cathode to enhance the interfacial properties, and investigation of the cell lifetime.

**Figure 10:**
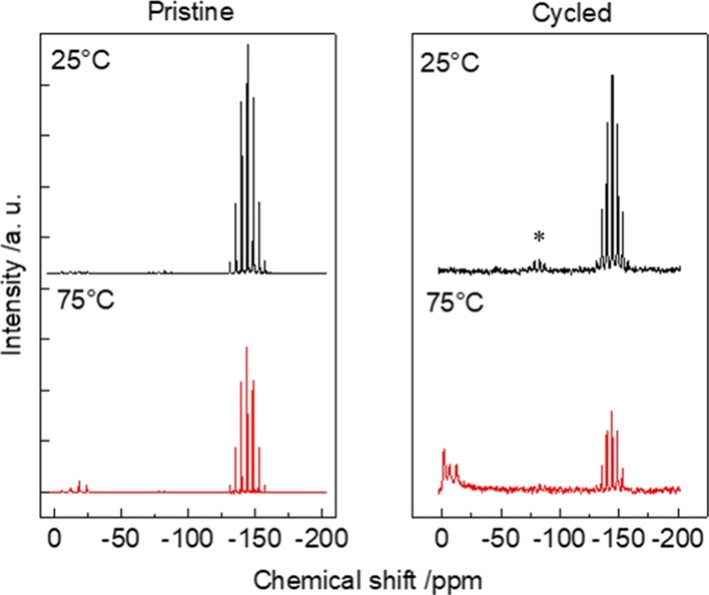
^31^P MAS-NMR spectra of the pristine sample (left panel) and of the cycled one (right panel) at two different temperatures. The stars indicate spinning sidebands.

The cross-linked gel electrolyte was finally disassembled from the cell and analyzed to evaluate the presence of degradation phenomena as a consequence of cycling. Figure 10 shows the ^31^P MAS-NMR spectra of the pristine sample (left panel) and the cycled one (right panel). In both cases, the spectra were obtained at 25 and 75 °C. The pristine sample at 25 °C (upper left) shows a multiplet at −146 ppm, which can be attributed to the PF_6_^–^ ions (J–J coupling in a AX_6_ system, with X being a 1/2-spin nucleus).^44^ The same sample treated at 75 °C (bottom left) shows traces of a triplet centered at ∼−15 ppm and of a doublet around −10 ppm. These two features were previously attributed to the intermediate degradation products (OPOF_2_)^−^ and (O_2_POF)^2–^, respectively.^39^ The cycled sample (upper right) shows the same PF_6_^–^ multiplet with a small spinning sideband feature (marked with stars) centered at −65 ppm. The presence of the sideband manifold is due to a wider pattern of anisotropic chemical shift, compatible with the presence of semimicroscopic susceptibility and/or with a more disordered structure, as already evidenced by the ^7^Li NMR spectra. Finally, the cycled sample treated at 75 °C (bottom, right) shows degradation products, attributed to (O_2_POF)^2–^ and PO_4_^3–^ at −10 and 0 ppm, respectively. It is noteworthy that the line widths of these two features are much larger (about 3 ppm at half height) because of the distribution of isotropic chemical shifts due to static disorder.^45^ The analysis of the spectra reported in Figure 10 shows that increasing the temperature to 75 °C leads to a partial decomposition of the electrolyte salt. This degradation is much stronger in the cycled sample, most likely because of the presence of impurities, for example, fragments of SEI, which can catalyze salt decomposition. It is noteworthy that the peaks of the degradation products of the cycled samples are characterized by higher homogeneous broadening, which reflects a greater structural disorder.

In general, the ^31^P-MAS-NMR results demonstrate the ability of the PEGDA-UPy cross-linked gel to stabilize LiPF_6_ at high temperatures by reducing the amount of fluorophosphates coming from PF_5_^–^-based decomposition reactions. Indeed, the intensity of the fluorophosphates and phosphate is mitigated with respects to what usually reported in the case of pure LP30 system.^46^ Such a functional gel acts as a PF_5_ scavenger, owing to the presence of nitrogen-based units (e.g., in ureidopyrimidinone) that complex PF_*x*_ through Lewis acid–base interactions, in nice agreement with what was reported in the case of amino-based additives specifically developed for LiPF_6_-based liquid electrolytes.^46,47^

## Conclusions

Highly cross-linked and uniform PEGDA-based gel electrolytes, blended with a self-healing polymer, were successfully synthetized in a very short time (30 min) by using only the reaction precursors to avoid other impurities sources. Gels with solid-like mechanical properties and a electrochemical stability window were obtained, even with high liquid electrolyte loading, namely for PEGDA-UPy 67, which exhibited G moduli of about 1 MPa and a voltage stability range higher than 5.5 V.

The PEGDA-UPy 67 gel also showed high room-temperature ionic conductivity and lithium transport number of 0.5 mS cm^–1^ and 0.6, respectively. The real applicability of such a quasi-solid electrolyte was proved in a Li/NMC full cell with stable cycling at least for 70 cycles at C/2 and a delivered specific capacity of 150 mA h g^–1^, comparable to that reported in the case of liquid electrolyte, over which the gel has significant advantages, in terms of interface stability, electrochemical stability window, and self-healing capabilities. Due to the optimal interplay among proper cross-linking density, polymer elasticity, and a suitable fraction of anions immobilized by the network, the PEGDA-UPy 67 showed a more stable Li stripping and plating process than the liquid electrolyte.

The proposed system gave evidence of the fast autonomous self-healing capability by means of dynamic multiple hydrogen bonding, which allowed for the full recovery of the ionic conductivity in case of loss of performance caused by physical damages.

## Supporting Information Available

^1^H NMR and FTIR spectra of polymer precursors; ^13^C MAS-NMR deconvolution of the carboxyl signal with peak assignment for the gel; TGA of the cross-linked gels and liquid electrolyte; DSC plots of pure PEGDA and gels; chronoamperometry and EIS plots for the determination of Li transport numbers in cases of PEGDA-UPy 67 and liquid electrolyte; best fit of the spin-lattice relaxation curve at 25 °C of the pristine gel and the same sample after galvanostatic cycling; ionic conductivity and optical microscopy images of gels; linear sweep voltammetry of PEGDA-UPy 67; EIS spectra of the symmetric cell Li|PEGDA-UPy 67|Li and the full cell Li|PEGDA-UPy 67|NMC; and SEM images of the Li metal electrode after stripping and plating experiments (PDF)
