# Supplementary material for: Cross-Linked Gel Electrolytes with Self-Healing Functionalities for Smart Lithium Batteries
Source: ACS Appl Mater Interfaces. 2022 Nov 10;14(46):51941–53. doi: 10.1021/acsami.2c15011 (PMC9706498; doi:10.1021/acsami.2c15011)
Supplement: Supplementary file 1 — am2c15011_si_001.pdf [file am2c15011_si_001.pdf]

Supporting Information

Cross-linked gel electrolytes with self-healing functionalities for smart Lithium batteries

S. Davino[a], D. Callegari[a], D. Pasini[a], M. Thomas[b], I. Nicotera[b,d], S. Bonizzoni[c], P. Mustarelli[c,d*] and E. Quartarone[a,d*]

a. *Department of Chemistry, University of Pavia, Via Taramelli 16, 27100 Pavia, Italy*
b. *Department of Chemistry and Chemical Technology, University of Calabria, Via P. Bucci, Rende, Cosenza 87036, Italy*
c. *Department of Materials Science, University of Milano Bicocca, Via Cozzi 55, 20126 Milano, Italy*
d. *GISEL - Centro di Riferimento Nazionale per i Sistemi di Accumulo Elettrochimico di Energia, INSTM via G. Giusti 9, Firenze 50121, Italy*

** Corresponding Authors:eliana.quartarone@unipv.it; piercarlo.mustarelli@unimib.it*

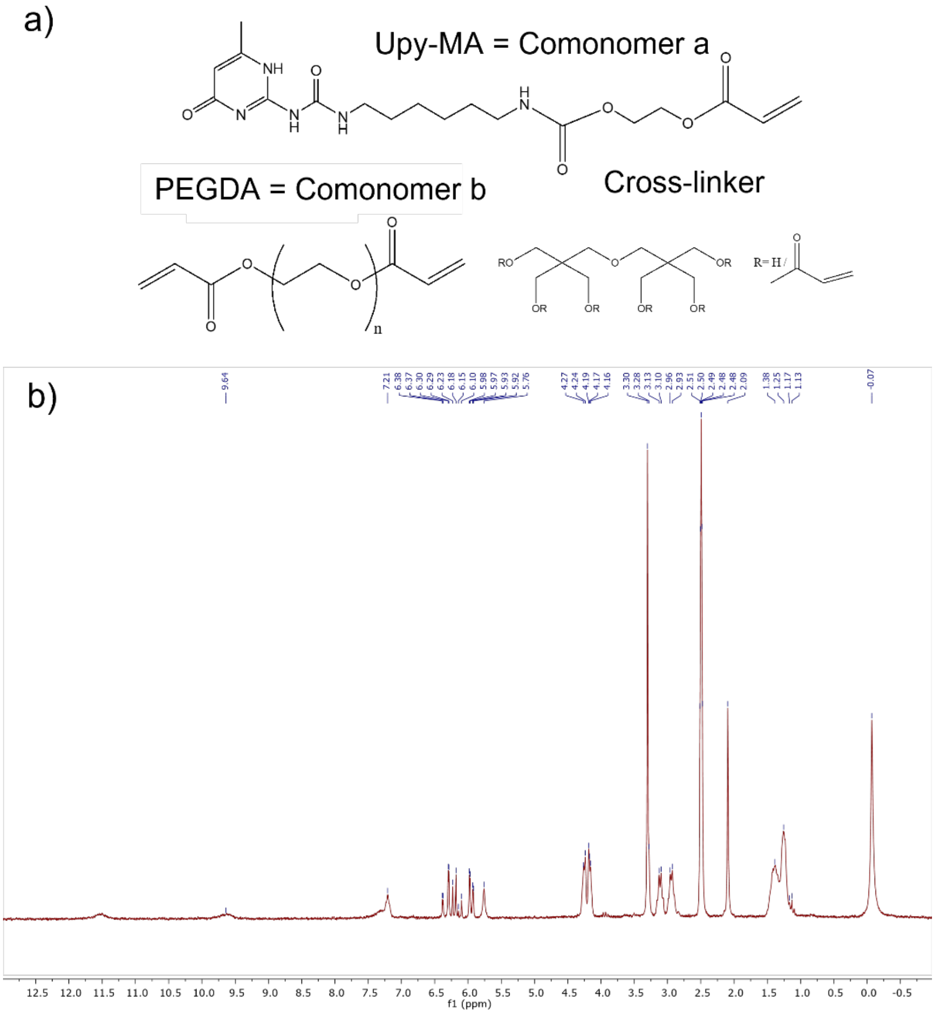

Figure S1. (a) Synthesis precursors of PEGDA-g-UPy 67;
(b) ^{1}H NMR of UPy-MA (comonomer *a*).

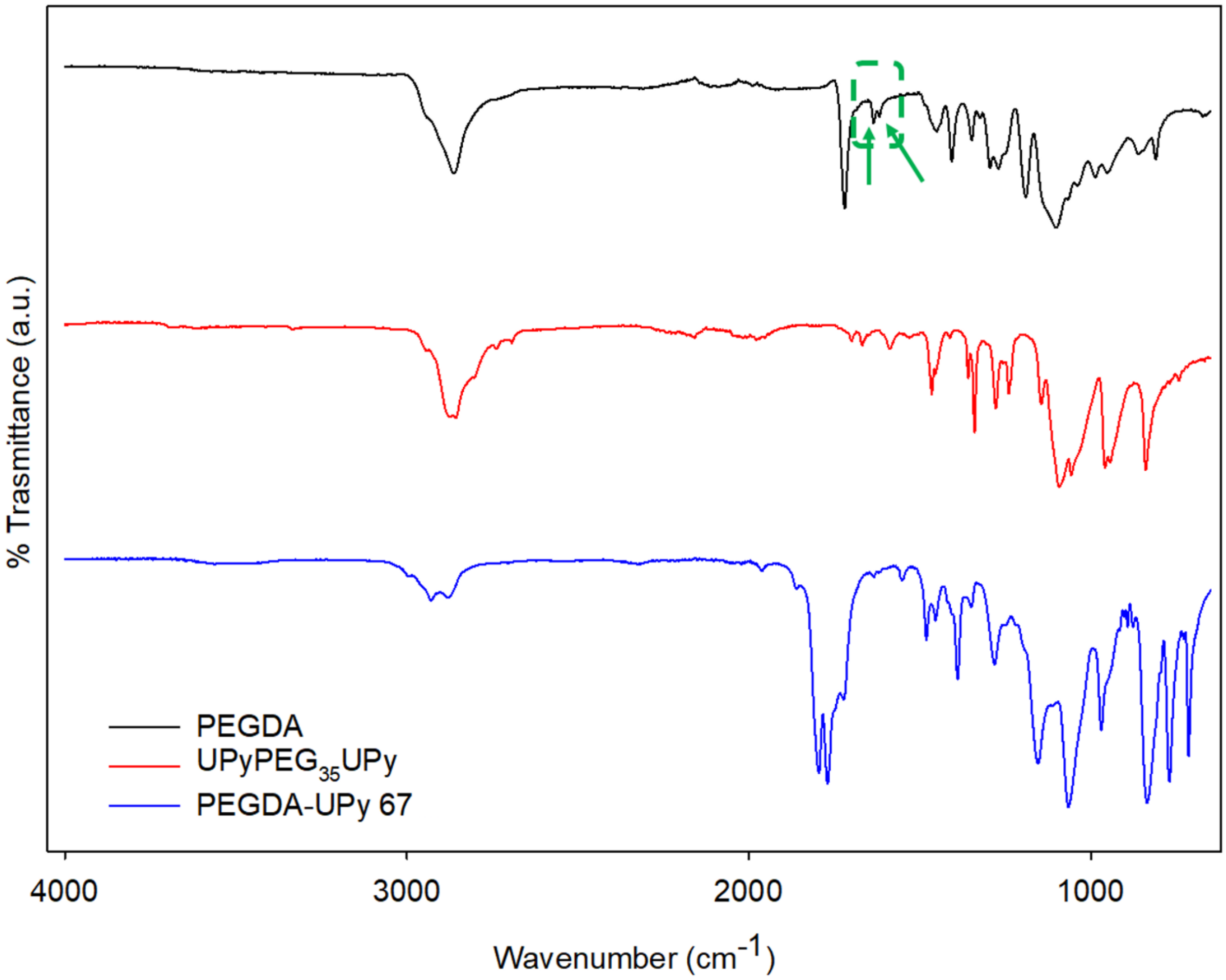

Figure S2. FTIR spectra of PEGDA comonomer, the self-healing units $UPyPEG_{35000}UPy$ and the final cross-linked gel electrolyte. The green arrows indicate the band corresponding to the vibrational modes of vinyl and acrylate double bonds involved in the polymerization reaction.

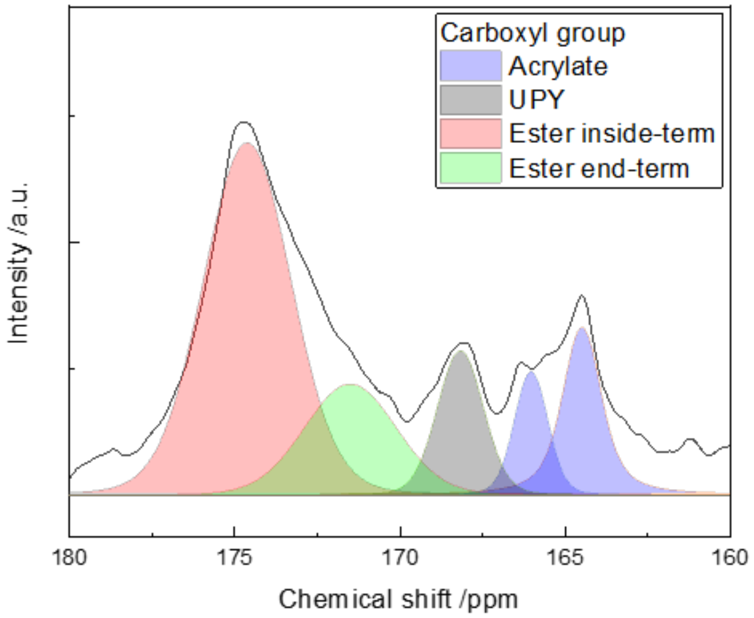

Figure S3. ^{13}C MAS-NMR deconvolution of the carboxyl signal with peaks assignment.

The degree of crosslinking (DC) is calculated as the ratio of the sum of acrylate carbonyl groups and the sum of ester carbonyl groups.

$$DC = \frac{Ester\ \ carboxyl\ signals}{Acrylate\ carboxyl\ signals} = \frac{6.78 * 10^7 + 2.07 * 10^8}{5.22 * 10^7 + 3.01 * 10^7} = 0.70$$

The branching of the linked structure could be calculated as the ratio of internal and end term of the ester carbonyl groups.

$$Branching = \frac{Inside - term\ ester\ carboxyl\ signals}{End - term\ ester\ carboxyl\ signals} = \frac{2.07 * 10^8}{6.78 * 10^7} = 3.0$$

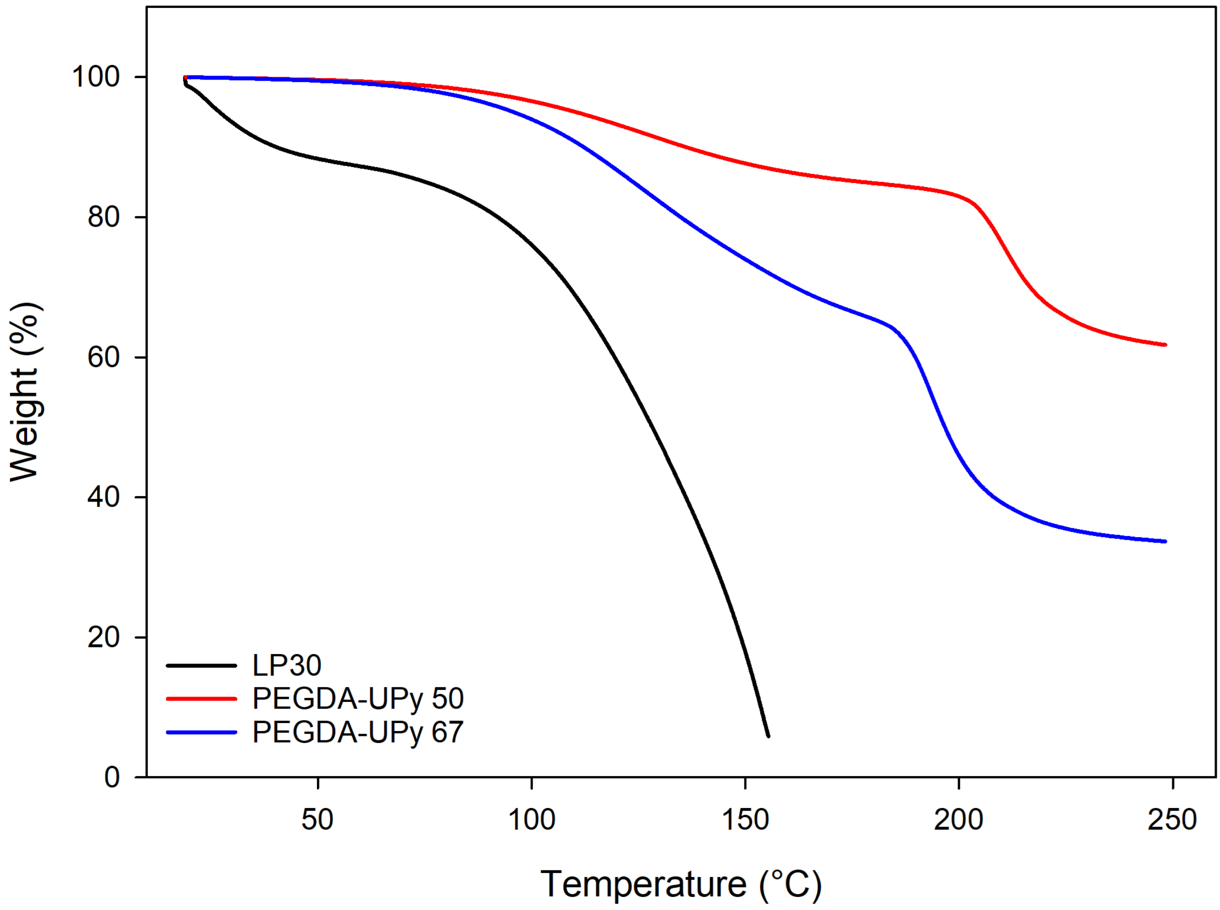

Figure S4. Thermogravimetry plots of the cross-linked gel electrolytes compared to the liquid electrolyte (LP30).

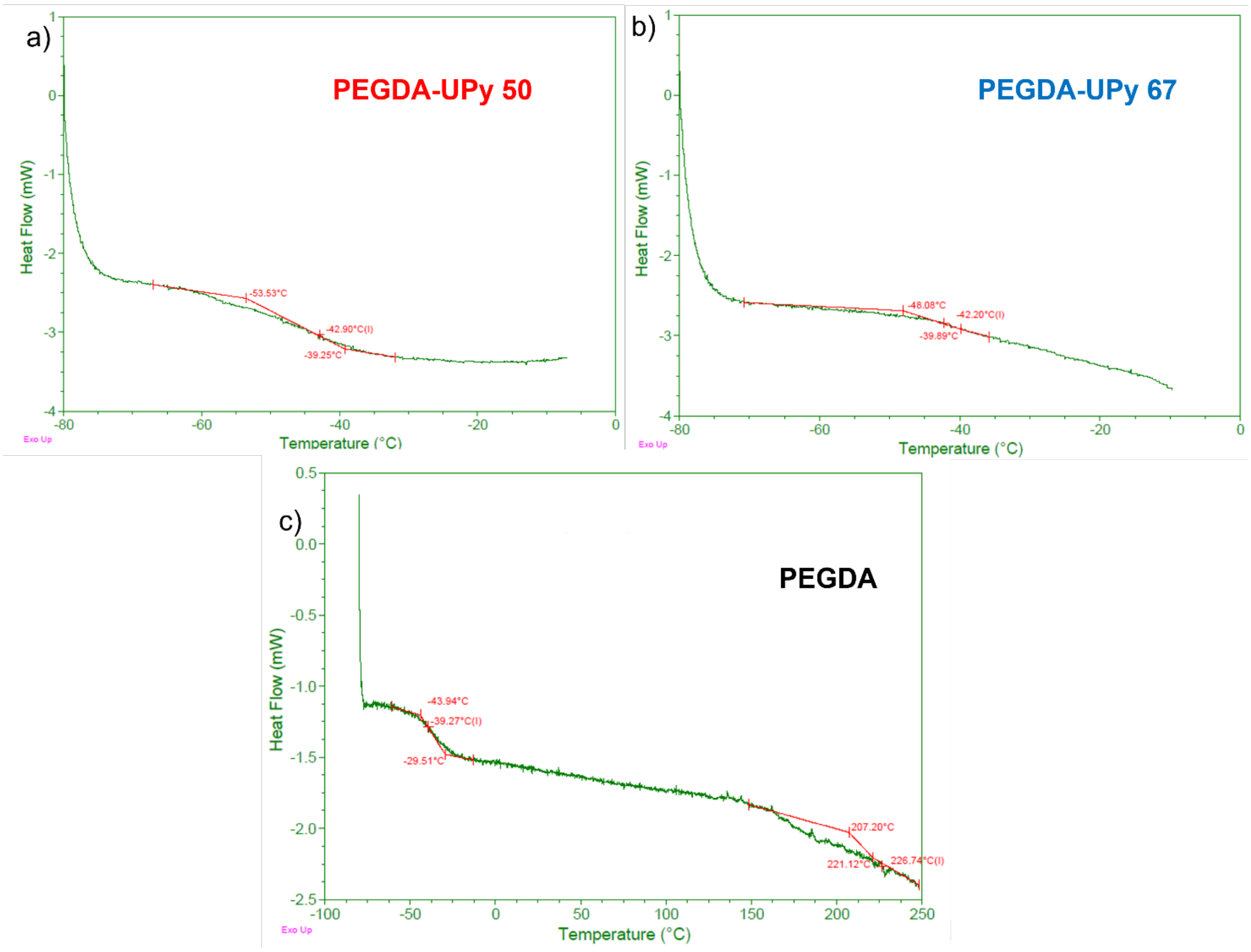

Figure S5. Branch of the DSC plots of pure PEGDA and gel electrolytes exhibiting the glass transition temperature, T_g.

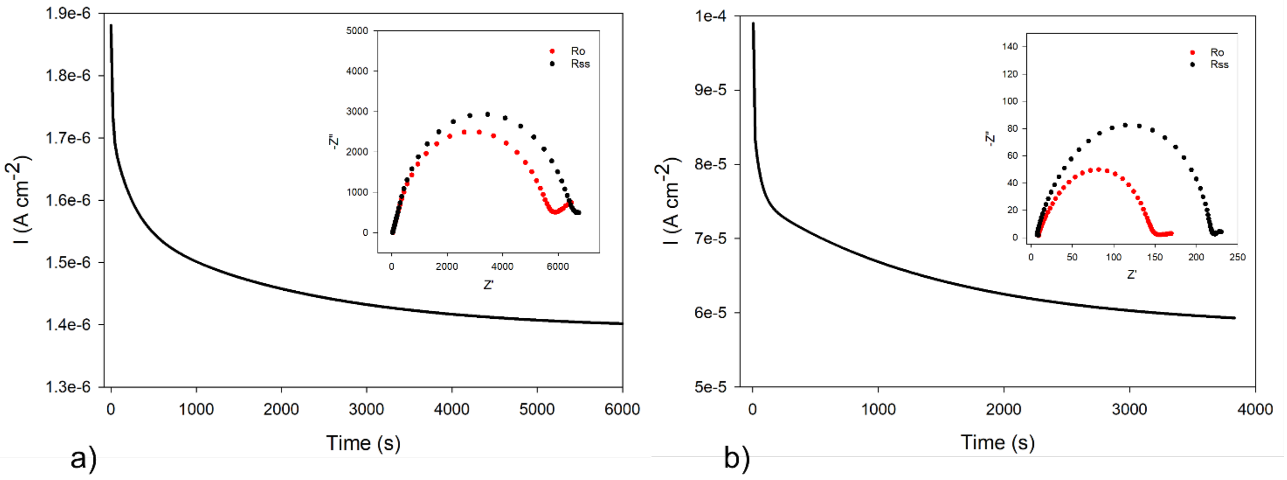

Figure S6. Chronoamperometry and electrochemical impedance spectroscopy plots for the determination of the Li transport numbers in case of PEGDA-UPy 67 (a) and LP30 (b).

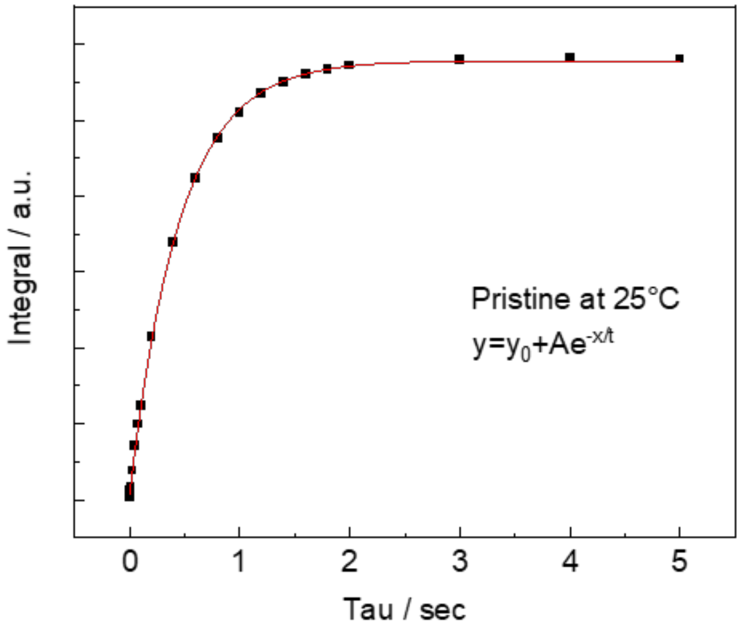

Figure S7. Best-fit (single exponential) of the spin-lattice relaxation curve at 25 °C of the pristine sample.

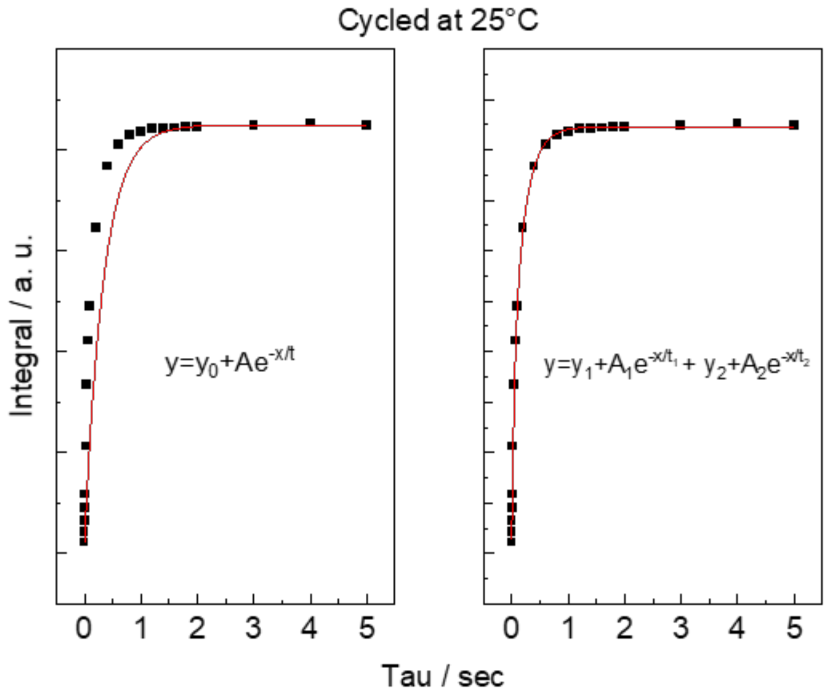

Figure S8. (left) best-fit (single exponential) of the spin-lattice relaxation curve at 25°C of the cycled sample; (right) best-fit (single exponential) of the spin-lattice relaxation curve at 25°C of the cycled sample.

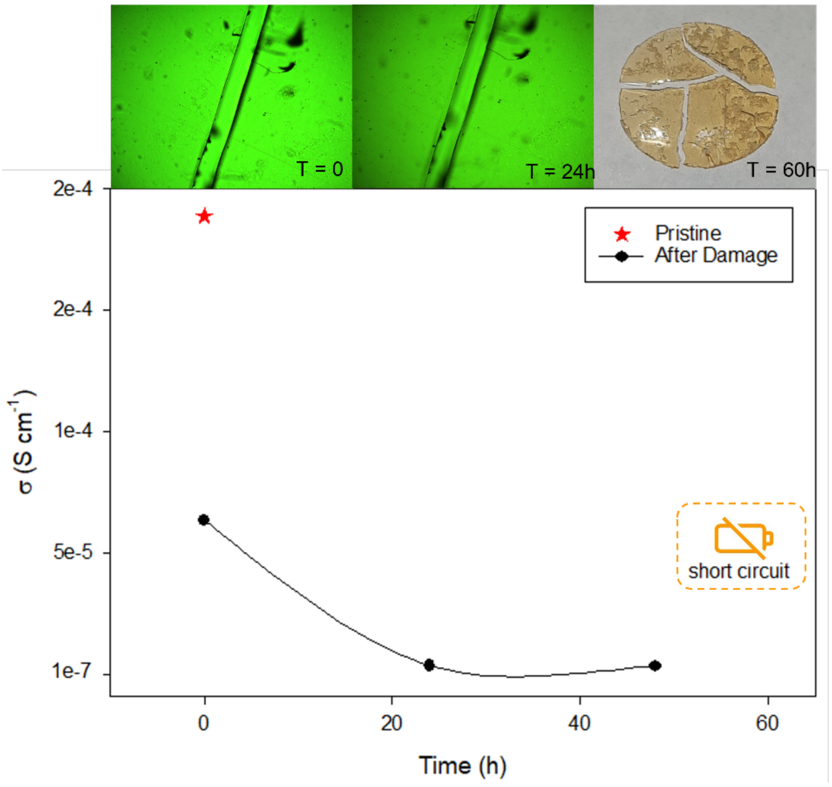

Figure S9. Room temperature ionic conductivity evolution with time in case of PEGDA-g-UPy after a deep cut over the whole film thickness (see images in the inset).

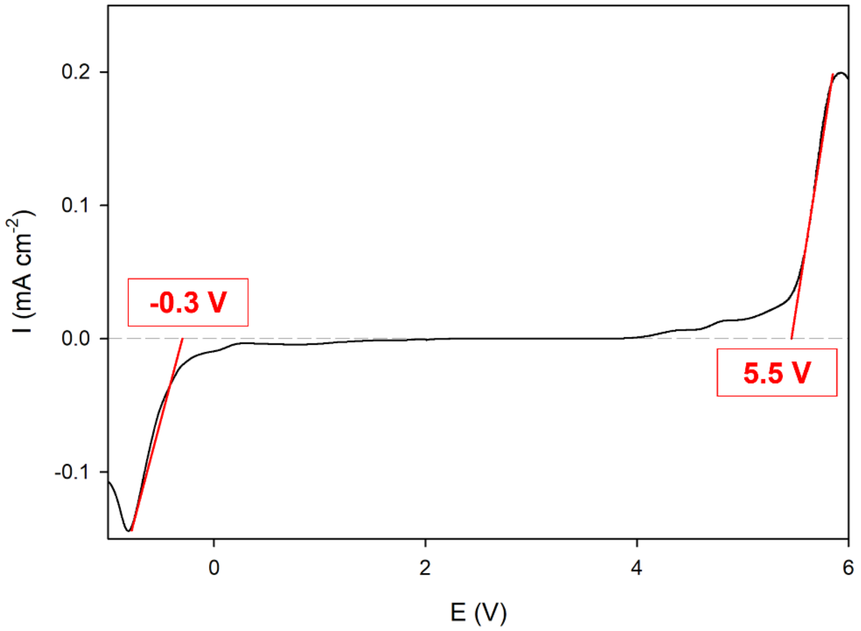

Figure S10. Linear sweep voltammetry at 0.25 mV s^{-1} in case of PEGDA-UPy 67 as electrolyte in a 3-electrode cell configuration (Li: counter-electrode and reference; C-coated Al: working electrode).

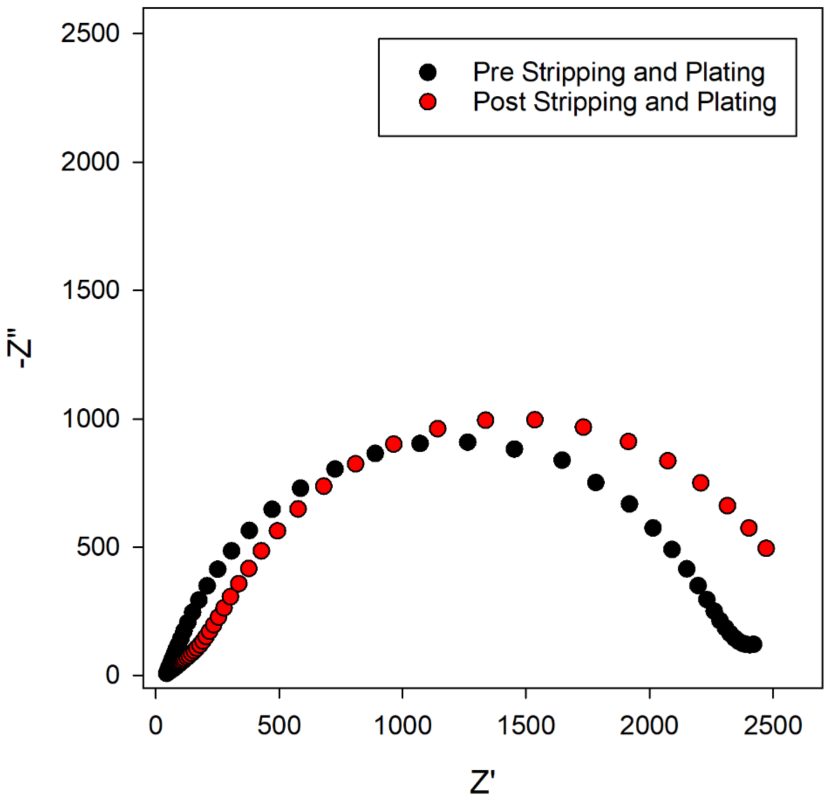

Figure S11. Nyquist plots collected by Electrochemical Impedance Spectroscopy on symmetric cell Li|PEGDA-UPy 67|Li at t=0 and t=230h

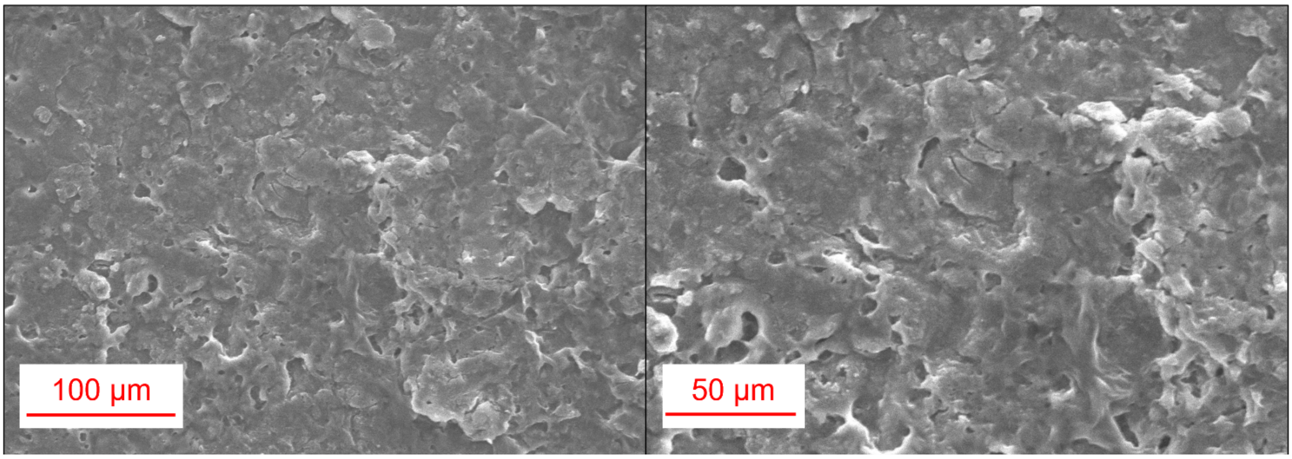

Figure S12. SEM images in top-view modes of Lithium Metal after the galvanostatic electrodeposition experiments.

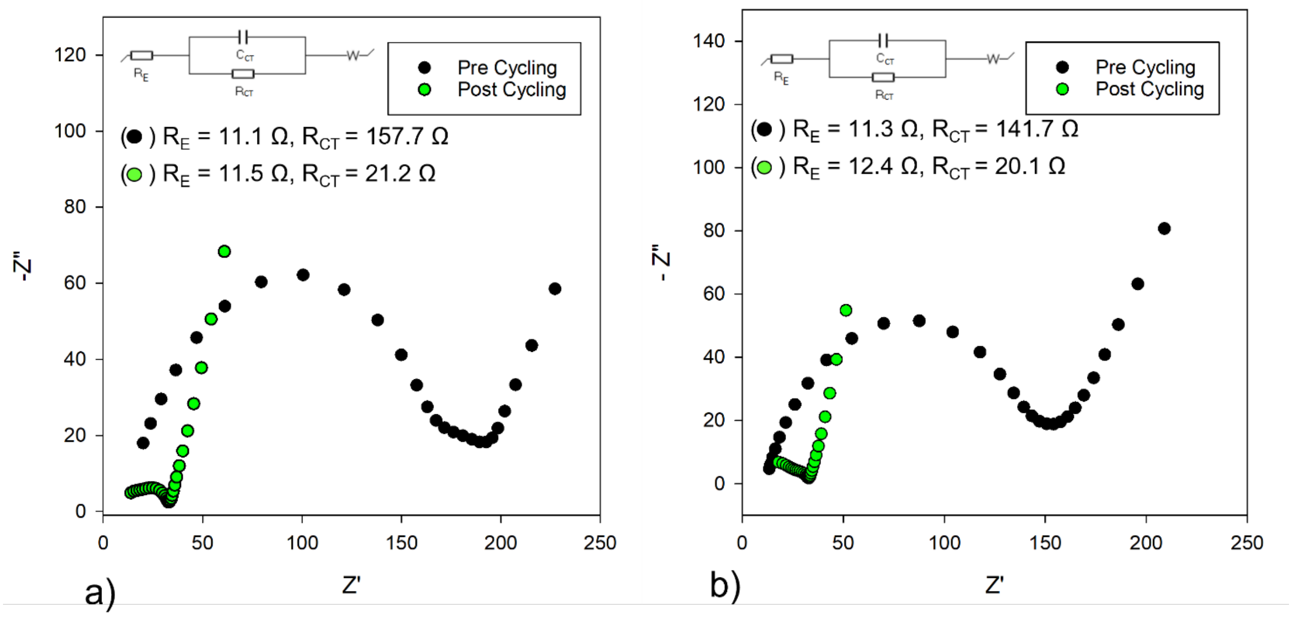

Figure S13. Nyquist plots collected by Electrochemical Impedance Spectroscopy on Li|PEGDA-UPy 67|NMC811 in case of rate performance at different C rates (a) and after cycling at C/2 (b)
